# Outcomes Affect Evaluations of Decision Quality: Replication and Extensions of Baron and Hershey’s (1988) Outcome Bias Experiment 1

**DOI:** 10.5334/irsp.751

**Published:** 2023-07-28

**Authors:** Sriraj Aiyer, Hoi Ching Kam, Ka Yuk Ng, Nathaniel A. Young, Jiaxin Shi, Gilad Feldman

**Affiliations:** 1 Department of Experimental Psychology, University of Oxford, UK; 2 Department of Psychology, University of Hong Kong, HK; 3 PhD student with the psychology department at DePaul University, US; 4 School of Psychology, South China Normal University, Guangzhou, CN; 5 Department of Psychology, University of Hong Kong, Hong Kong SAR, HK

**Keywords:** outcome bias, evaluation of decisions, responsibility, cognitive biases, pre-registered replication, open science

## Abstract

Outcome bias is the phenomenon whereby decisions which resulted in successful outcomes were rated more favorably than when the same decisions resulted in failures. We conducted a pre-registered replication and extension of Experiment 1 (original’s: *N* = 20) from the classic Baron and Hershey (1988) with an online Amazon Mechanical Turk sample using CloudResearch (*N* = 692), switching from a within-participants design in the original experiment to a between-participants design. We tested outcome bias by measuring participants’ ratings of the quality of decisions in medical scenarios. For the replication (pre-registered) part of the study, we successfully replicated signal and direction of the outcome bias (original: *d_paired_* = 0.21 – 0.53; replication: *d_independent_* = 0.77 [0.62, 0.93] to 1.1 [0.94, 1.26]), and even for participants who stated that outcomes should not be taken into consideration when evaluating decisions (*d* = 0.64 [0.21, 1.08]). For the extension part of the study, we found differences, dependent on outcome types, in evaluations of the perceived importance of considering the outcome, the perceived responsibility of decision-makers, and the perception that others would act similarly given the choice by outcome type. Materials, data, and code are available on Open Science Framework (OSF): https://osf.io/knjhu/.

## Background

Research by Baron and Hershey (1988) demonstrated that outcomes tend to affect evaluations of the quality of decisions associated with those outcomes, for both evaluations of one’s own decisions and evaluations of others’ decisions (Baron & Hershey, 1988). This means that evaluations of decision quality are often revised after the consequences of a decision are made known, in that the same decision is evaluated post hoc to be more positive if it resulted in a positive outcome than if it resulted in a negative outcome. In an unbiased situation, the judge processes only the information available to the decision-maker at the time of a decision. Considering that outcomes may not be related to the quality of the decision, outcome information should not affect the judgment of the decision in most cases.

Our first goal was to conduct an independent pre-registered and well-powered replication of a classic article on outcome bias. Our second goal was to add and examine extensions that investigated whether outcome bias is informed by perceptions about the importance of outcomes in decisions: the responsibility of decision-makers or the social norms about using outcomes to evaluate the quality of decisions.

## Chosen Study for Replication: Baron and Hershey (1988)

Baron and Hershey (1988) demonstrated outcome bias using five experiments. In their Experiment 1, participants were presented with 15 cases of medical decisions in a within-participants design. They were asked to evaluate each decision and explain their reasoning. The cases only differed by two factors: (1) the decision-maker (self or other) and (2) the outcome (success or failure). They showed that participants gave higher ratings to medical decisions that resulted in positive outcomes compared to medical decisions that resulted in negative outcomes, despite the decisions themselves being identical outside of their outcome. In addition, outcome bias occurred despite participants indicating that they believe outcomes should not impact their judgment.

We found the target article to be good a target for replication because the original study was based on a sample size of 20, which resulted in effect size estimates with relatively wide confidence intervals. A larger sample would hence allow us to obtain an estimate of effect size with higher precision. Moreover, in the original study’s within-participants design, some participants reported that they gave the same evaluation to decisions regardless of their outcomes because they remembered the rating given to the previous case with a different outcome. The original authors noted (Baron & Hershey, 1988) that a within-participants design makes it easier to distinguish small effects from random noise, but it does so at the cost of effect size magnitude due to participants’ recall of previous responses. We therefore aimed to investigate whether results would generalize to a between-participants design. Between-participants designs can clear up potential issues around subject awareness of the experimental manipulation and whether outcome bias persists across changes of intentionality (Jamison, Yay & Feldman, 2020). A between-participants design is also more analogous to real-life situations where outcome bias could arise. Thus, a demonstration of the effect, even in a between-participants design, would increase confidence in the robustness and applicability of the phenomenon, ensuring that the target’s specific outcome bias experimental design is not being driven by demand characteristics and aspects of intentionality inherent to a within-participants design.

Outcome bias has been used as a foundation for a wide variety of subsequent research, and the target article has been cited well over 1,000 times. The wealth of research following the findings by Baron and Hershey (1988) demonstrates their importance to psychology and other fields, such as metascience (Emerson et al., 2010), medical decision-making (Savani & King, 2015), and social psychology (Mackie & Ahn, 1998). However, the sample size of the original study was too small to obtain a realistic estimate of the outcome bias’s effect size. Hence, we felt it important to revisit these findings to assess their reproducibility and replicability and to update these findings to obtain more current effect size estimates and, indeed, to see if an effect is still observable with a larger sample. Ensuring the robustness of the original study’s findings would in turn bolster subsequent research that builds upon Baron and Hershey’s research.

## Replication Closeness Evaluation

We aimed to conduct a replication and extension of Experiment 1 from Baron and Hershey (1988). Based on LeBel et al.’s (2018) criteria for the evaluation of replications (Table 1), we classified our experiment as a ‘close to far’ replication of the original study. Many of the features were the same or similar, which we felt warranted the categorization of ‘close’, and yet the evaluation criteria we used made no reference to the impact of shifting study design from a within-participants to a between-participants design; to accommodate for that change, we summarized that as ‘close to far’. We note that other replications with similar adjustments from a within-participants to a between-participants design have been classified as a ‘close replication’ (Jamison, Yay & Feldman, 2020).

**Table 1:** LeBel et al. (2018) replication taxonomy for this replication study to classify the methodological similarity to the original study from Baron and Hershey (1988). Each facet of the experimental design within Baron and Hershey (1988) and our current study are compared to each other for similarity, with any key differences between the two described.


DESIGN FACET	SAME/DIFFERENT TO ORIGINAL PAPER	NOTES

Effect/hypothesis	Same	

IV construct	Same	

DV construct	Same	

IV operationalization	Same	

DV operationalization	Same	

Population (e.g., age)	Different	Original study recruited undergraduates.

IV stimuli	Same	

DV stimuli	Similar	Original DV stimuli used, with added extensions.

Procedural details	Different	Study conducted via Qualtrics, changed from within-participants to a between-participants study design.

Physical setting	Different	Study conducted online rather than in person.

Contextual variables	Different	

Overall	Close to far replication	


## Deviations from the Baron and Hershey (1988)

We deviated from the original study in a number of ways. A major deviation is that we shifted from the original study’s within-participants design to a between-participants design in order to investigate the generalizability of outcome bias and adapt it to a more realistic situation in which persons are only exposed to either a positive or a negative outcome of a decision rather than being presented with both outcomes. We used a different physical setting, as the original study was conducted in person, whereas we conducted the study online with participants recruited from labor markets, and so our study population was different to that of the original study, which recruited only undergraduate students from the University of Pennsylvania (UPenn). In the target article, the claims made were not about a specific population (UPenn students); therefore, we assumed broader generalizability to other populations. In addition, we also added extensions with additional dependent variables and comprehension check questions.

## Extensions: Outcome Importance, Responsibility, and Perceived Norms

To go beyond the replication, and extend the original findings, we investigated participants’ perceptions of outcome bias.

Baron and Hershey (1988) investigated whether the consideration of the importance of outcome information influenced outcome bias. However, this analysis only included eight participants. We aimed to test the idea that the perceived importance of outcome information at least partially explains the outcome bias by investigating outcome importance as a variable that mediates the relationship between outcome and decision judgment. Alternatively, Baron and Hershey (1988) noted that the participants may have thought that the physicians were more responsible for failed outcomes than patients. This could be due to the expectations of care and transparent disclosure of errors that may befall patients (Finkelstein et al., 1997). In addition, the ideal of patient empowerment (patients taking more responsibility and initiative for their own health) is still relatively nascent (Ishikawa, Hashimoto & Kiuchi, 2013). We therefore added an extension examining perceptions of decision-maker responsibility (physician or patient) as a mediator of outcome bias.

Lastly, the perception of how others would act in the same situation may relate to outcome bias. Given that outcome bias leads people to inappropriately consider outcome information when judging decisions, successful outcomes may lead people to think that most people would choose similarly, and failed outcomes may lead people to think the alternative is what most people would choose. This biased perception, due to the outcome information, may at least partially explain why people are affected by outcome information. According to norm theory (Kahneman & Miller, 1986), individuals use available information to reconstruct perceived norms regarding what is normal or, in this case, what others would do when faced with a certain decision. In this case, exposure to a successful or unsuccessful outcome would impact the perceived norms of how other individuals would act under similar circumstances. If it is considered the socially accepted course of action to judge decisions by their outcome, this explains why such a bias may present itself. We would hence have an insight into whether such an outcome bias is conscious or unconscious. Both our replication and extension hypotheses are provided in Table 2.

**Table 2:** Baron and Hershey (1988) replication and extension hypotheses.


#	HYPOTHESIS

Original	

1	Decisions that resulted in successful outcomes are rated as better than decisions that resulted in failed outcomes.**

2	Participants who report thinking that judgments should not be based on outcomes demonstrate an outcome bias.**

Extensions	

3a	Successful outcomes are rated higher on outcome importance than failed outcomes.*

3b	Perceived outcome importance partially accounts for (mediates) outcome bias.

4a	Failed outcomes are rated as higher perceived level of responsibility of the decision-maker than in successful outcomes.*

4b	Perceived decision-maker responsibility partially accounts for (mediates) outcome bias.

5a	Decisions resulting in failed outcomes are perceived as less normative than decisions resulting in positive outcomes.

5b	Perceived norms partially account for (mediates) outcome bias.


*Note*: ** Pre-registered hypotheses in both OSF pre-registrations. * Pre-registered hypotheses in one pre-registration. Two pre-registrations were created by independent analysts. Refer to Table 3 for details on the divergence between pre-registrations.

**Table 3:** Comparison between the two crowdsourced pre-registrations.


HYPOTHESIS/ANALYSIS	H. C. K. PRE-REGISTRATION	K. Y. N. PRE-REGISTRATION

Original hypotheses	Included	Included

Extension hypotheses	Included	Not included

2 × 2 ANOVAs on DVs	Included	Not included

Power analyses	Included	Included

Exclusion criteria	Included	Included


*Note*: H. C. K.’s pre-registration is available on OSF (https://osf.io/pwgq4), and K. Y. N.’s pre-registration file is available here: https://osf.io/ydxg7. Our aim was to crowdsource the pre-registrations by having two coauthors independently analyze the target article and plan analyses. We aimed to address both by following the strictest, most conservative combination of the two. The Qualtrics survey was included in the pre-registration and is available on OSF: https://osf.io/q4xbf (exported Word file) and https://osf.io/vfw38 (QSF Qualtrics import file).

## Pre-Registration and Open-Science

We crowdsourced pre-registrations via two coauthors working independently in tackling the analysis and reproduction of Baron and Hershey’s (1988) methods and analyses. There were very few differences between the two pre-registrations, probably due to the simple design of the target article. We therefore pre-registered both together, aiming for addressing the most conservative combination of the two. Through this process, we aimed to restrict our researchers’ degrees of freedom and benefit from different views on what should be pre-registered.

We then pre-registered the experiment on OSF (https://osf.io/czha8/), and data collection was launched later that week. Open-science details and disclosures, power analyses, and all materials used are detailed in the supplementary materials. Materials, data, and code were made available on OSF: https://osf.io/knjhu/.

All measures, manipulations, and exclusions conducted for this investigation are reported. All studies were pre-registered, with power analyses reported in the supplementary, and analyses were only conducted after all data had been collected.

## Method

### Participants

In total, 707 participants were recruited online from Amazon’s Mechanical Turk (MTurk) using CloudResearch/TurkPrime (Litman et al., 2017). Four pre-registered exclusion criteria were adopted to maintain data quality. These exclusion criteria included (1) failure to complete the survey (i.e., not completing all study questions); (2) low English proficiency (rating of less than 5 on a self-rated 1–7 scale for the question ‘How would you generally rate your understanding of the English used in this study?’); (3) self-reported lack of seriousness when completing the survey (rating of less than 4 on a self-rated 1–5 scale for the question ‘How serious were you in filling out this questionnaire?’); and (4) participants who correctly guessed the hypothesis of the study. We ran this study alongside a few other unrelated studies within the same Qualtrics survey (with the studies presented in a random order), and hence the question on English proficiency was shared among these studies (specifically, with Ziano et al., 2023).

After excluding participants who did not complete the survey, 705 participants remained (2 excluded). After considering the other three criteria, 692 participants remained (13 excluded) (364 female; *M*_age_ = 38.9, range: 19–78). Exclusions had little impact on the results; a summary of the results from an analysis on the complete dataset is available in the Supplemental Materials. All questions were forced response; therefore, there was no missing data.

We initially conducted a power analysis of the effects for the differences between conditions 1 (Physician Success) and 2 (Physician Failure) and between conditions 3 (Patient Success) and 4 (Patient Failure) of Experiment 1 in Baron and Hershey (1988). Using GPower, we determined that at least 239 participants were needed to achieve 95% power to detect the minimum effect of *dz* = 0.21 with an alpha of 0.05 for the smaller effect of the two contrasts, one-tailed, for a within-participants design (see Supplemental Materials, page 7). However, given the very conservative estimate of the effect, and our shifting the design to a between-participants design with uncertainty about how this would impact effects, we decided to multiply the sample size by a factor of 2.5 in line with the recommendation from Simonsohn (2015), aiming for ~600 participants after exclusions, recruiting ~700. Our post-exclusion analysis below resulted in 692 participants, and our sensitivity analysis indicated that a between-participants design allows the detection of Cohen’s *d* = 0.25 (one tail). The sensitivity analysis and power curve are available in the Supplemental Materials.

Below we detail the stimuli and questions shown to the participants in the order that they were presented, which was fixed (and not randomized) for all participants. We reported a correlation matrix of all dependent variables in the Supplemental Materials, where some measures were found to be weakly correlated.

### Outcome Bias Manipulation

A summary of the stimuli and manipulations used are provided in Table 4. Participants were randomized between the four conditions as per a 2 (Decision-maker: Physician or Patient) × 2 (Outcome: Success or Failure) design. All four decision conditions began with the same description of a medical case shown to participants:

A 55-year-old man had a heart condition. He had to stop working because of chest pain. He enjoyed his work and did not want to stop. His pain also interfered with other things, such as travel and recreation. A type of bypass operation would relieve his pain and increase his life expectancy from age 65 to age 70. However, 8% of the people who have this operation die from the operation itself.

**Table 4:** Replication study experimental design.


**IV1: Outcome [Between]**	IV1: Outcome manipulation Outcome: Success	IV1: Outcome manipulation Outcome: Failure
**IV2: Decision-maker [Between]**	Manipulation: ‘The operation succeeded.’	Manipulation: ‘The operation failed.’

**IV2: Decision-maker manipulation condition A** Decision-maker: Patient Manipulation: Participant was told ‘the patient decided to go ahead with the operation’.	**Dependent variables** Decision quality: ‘Please evaluate the physician’s/patient’s decision on a scale from 3 (Clearly correct and the opposition decision would be inexcusable) to –3 (Incorrect and inexcusable).’ Perceived outcome importance: ‘Do you think you should take the outcome into account in evaluating the decisions? Please rate on a scale from 1 (Definitely not) to 5 (Definitely yes).’ Perceived responsibility: Item: ‘Rate the level of responsibility of the patient for the decision made to go ahead with the operation on a scale from 1 (No responsibility) to 7 (Full responsibility).’ Perceived norms: Item: ‘Do you think that most people in this situation would decide to go ahead with the operation? Please rate on a scale from 1 (Definitely not) to 5 (Definitely yes).’	

**IV2: Decision-maker manipulation condition B** Decision-maker: Physician Manipulation: Participant was told ‘his (the patient’s) physician decided to go ahead with the operation.’		


The wording at the end of the scenario description manipulated who decided whether to proceed with the operation and outcome of the operation. This was worded as follows (with the modified text shown in square brackets):

[The patient/His physician] decided to go ahead with the operation. The operation [succeeded/failed]. Evaluate the [patient’s/physician’s] decision to go ahead with the operation.

### Measures

#### Comprehension Checks

An adjustment made to the original study was the inclusion of comprehension checks. As the manipulations were made by changing key pieces of information in the scenario, we had to ensure that participants paid attention to key details such as the decision-maker and outcome. This allows us to be confident that these aspects were driving any observed differences in decision quality (especially given our between-participants design). Participants were required to view the instructions and answer all questions correctly before they could proceed with the study in order to ensure they were fully aware of the stimuli shown.

We included four comprehension checks that participants had to answer correctly before proceeding to the next page to the evaluations: ‘Who made the decision to go ahead with the operation?’; ‘What percentage of people who had the operation died from the operation?’; ‘Which of the following is an advantage of the operation?’; and ‘Was the operation successful?’

#### Decision Quality

Participants were asked to evaluate each decision on a 7-point Likert type scale: ‘Please evaluate the physician’s/patient’s decision.’ Participants then responded on a scale from 3 (Clearly correct and the opposition decision would be inexcusable) to –3 (Incorrect and inexcusable). Participants were then asked to briefly explain their evaluations.

#### Perceived Outcome Importance

Participants indicated whether they thought that they should have taken the outcome into account when evaluating the decision: ‘Do you think you should take the outcome into account in evaluating the decisions?’ (1—Definitely not; 5—Definitely yes).

#### Perceived Responsibility

Participants rated the level of responsibility of the decision-maker (physician/patient): e.g., ‘Rate the level of responsibility of the patient for the decision made to go ahead with the operation.’ (1—No responsibility; 7—Full responsibility).

#### Perceived Norms

Participants rated perceived norms: ‘Do you think that most people in this situation would decide to go ahead with the operation?’ (1—Definitely not; 5—Definitely yes).

## Results

Descriptive statistics of each dependent measure are provided in Table 5. All analyses were conducted using the software R (R core Team, 2017), version 4.1.3. We provided the analysis scripts and data files in the OSF folder (https://osf.io/knjhu/).

**Table 5:** Means and standard deviations for each measured variable for all conditions.


	SUCCESS					FAILURE				
	
	PHYSICIAN(*n* = 173)		PATIENT(*n* = 171)		PHYSICIAN(*n* = 172)		PATIENT(*n* = 176)	
			
	*M*	*SD*	*M*	*SD*	*M*	*SD*	*M*	*SD*

Evaluation	1.81	0.84	1.76	0.79	0.45	1.55	0.90	1.36

Outcome importance	4.49	0.82	4.40	0.77	4.13	1.04	4.28	0.91

Responsibility	5.88	1.02	6.13	0.97	5.23	1.36	5.78	1.21

Act the same	4.03	0.68	4.06	0.62	3.71	0.87	3.86	0.75


We conducted an analysis of variance (ANOVA) for each dependent variable with outcome type (success/failure) and decision-maker (physician/patient) as between-participants factors. We report 90% confidence intervals, as they exclude 0 for one-sided *F*-tests, unlike 95% confidence intervals (Lakens, 2013). In addition, Welch’s independent samples, one-tailed *t*-tests were used to test specific hypotheses by outcome type. We use one-tailed tests given that we had clear hypotheses and aimed to replicate and confirm clear predictions reported in the target article, though we note that the relatively strong effects in support of outcome bias hold for two-tailed tests.

### Confirmatory (Pre-Registered) Results

#### Replication: Decision Quality

We conducted three analyses to examine the replication hypotheses. First, to test outcome bias, we ran a 2 outcome type (success/failure) × 2 decision-maker (physician/patient) between-participants ANOVA. We found support for a main effect of decision-maker over perceived decision quality (*F*(1, 688) = 4.73, *M*_diff_ = 0.20, *p* = .030, Cohen’s *f* = .08, 90% CI [0.02, 0.15]). Patients’ decisions were evaluated as higher quality (*n* = 347, *M* = 1.33, *SD* = 1.20) than that of physicians’ (*n* = 345, *M* = 1.13, *SD* = 1.42; with Bonferroni corrections: *p* = .090).

Furthermore, we found support for a main effect of outcome type (*F*(1, 688) = 152.37, *M*_diff_ = 1.11, *p* < .001, Cohen’s *f* = .47, 90% CI [0.40, 0.53]). Decisions resulting in positive outcomes were evaluated as higher quality (*n* = 344, *M* = 1.78, *SD* = 0.81) compared to those resulting in negative outcomes (*n* = 348, *M* = 0.68, *SD* = 1.47; with Bonferroni corrections: *p* < .001).

We concluded that these findings indicate a successful replication of the phenomenon in terms of direction and signal that supports the predictions and the aggregate findings of the original’s experiment. We observed larger effect size for the specific scenarios our replication was focused on, yet we note the need for caution in comparing effects from different study designs.

**Figure 1:**
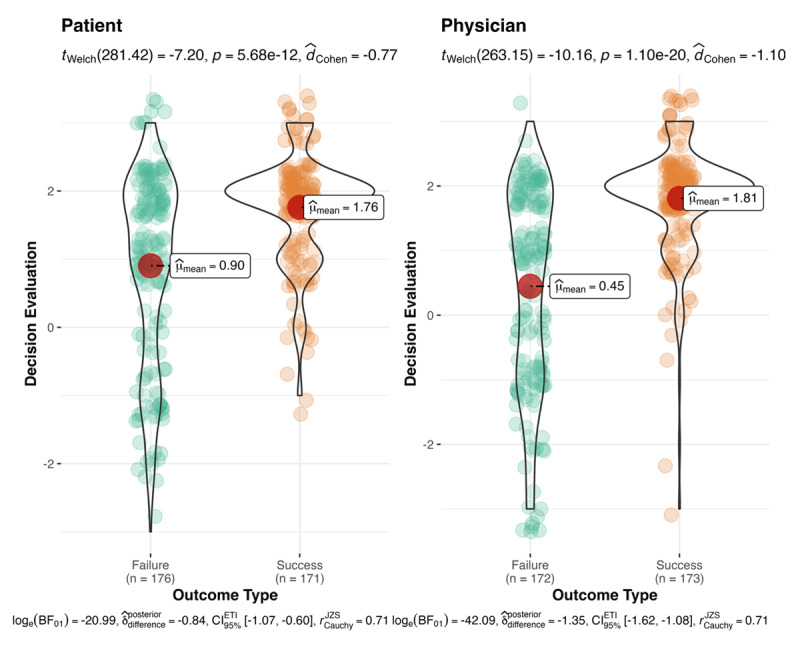
Decision quality evaluations: decision-maker and outcomes. **Note:** The effect of outcome type (success vs. failure) and decision-maker type (physician vs. patient) on evaluations of decisions. Successes were rated as more correct than failures. Patients were rated more correct on average regardless of the outcome compared to physicians. Outcome type and decision-maker type interacted to account for decision evaluations. Physicians’ decisions were evaluated as less correct than patients’ decisions when the outcome was a failure but equally as correct when the decision resulted in a success. Bayes factors are reported as per the built-in function within the ggstatsplot package in R.

We further tested and found an interaction of outcome type and decision-maker type (*F*(1,688) = 7.9, *p* = .005, Cohen’s *f* = .11, 90% CI [0.04, 0.17]; with Bonferroni corrections: *p* = .015). Follow-up post hoc Welch’s *t*-tests showed that physicians (*t*(263.15) = 10.16, *M*_diff_ = 1.36, *p* < .001, Cohen’s *d* = 1.10, 95% CI [0.94, 1.26],) and patients (*t*(281.42) = 7.20, *M*_diff_ = 0.86, *p* < .001, Cohen’s *d* = 0.77, 95% CI [0.62, 0.93]) were evaluated as more correct when they resulted in success than when they resulted in failure, confirming an outcome bias effect for both the physician and patient conditions. However, physicians’ decisions were evaluated as lower quality than patients’ decisions (*t*(338.62) = –2.91, *M*_diff_ = 0.86, *p* = .004, Cohen’s *d* = –0.31, 95% CI [–0.46, –0.16]) when they resulted in failure, but physicians and patients were evaluated as similarly correct when they resulted in success (*t*(341.10) = 0.56, *M*_diff_ = 0.86, *p* = .58, Cohen’s *d* = 0.06, 95% CI [–0.09, 0.21]; see Figure 1). We summarized participants’ justifications for their evaluation of the decision in Table 6.

**Table 6:** Evaluation justifications’ ratings.


JUSTIFICATION	PHYSICIAN SUCCESS	PHYSICIAN FAILURE	PATIENT SUCCESS	PATIENT FAILURE	TOTAL

Outcome	45	19	28	24	116 (16.5%)

Ethical concerns	16	29	3	0	48 (6.8%)

Others	108	123	139	153	523 (35.9%)

Unclear	8	4	6	0	18 (2.6%)

Total	177	175	176	177	705


#### Comparing Replication to Original: Equivalence

We summarized a comparison of the findings reported by the target article and our replication in Table 7.

**Table 7:** Comparison of effects between the target article and our replication.


DECISION-MAKERS	ORIGINAL EFFECT SIZE ESTIMATE (*d_paired_*) AND 95% CONFIDENCE INTERVALS	REPLICATION EFFECT SIZE (*d_independent_*) AND 95% CONFIDENCE INTERVALS	REPLICATION INTERPRETATION (LEBEL ET AL., 2019)

Patient	0.21 [–0.23, 0.66]	0.77 [0.62, 0.93]	Signal and same direction

Physician	0.53 [0.06, 0.99]	1.10[0.94, 1.26]	Signal and same direction

Aggregate of all scenarios	0.90 [0.37, 1.42]		


*Note*: The effect for the original is for paired samples, whereas our replication is for independent samples and should therefore be interpreted with caution.

We conducted a follow-up equivalence test to try and compare the outcome bias effects in Baron and Hershey (1988) and our replication. We used a two one-sided tests (TOST) procedure (Lakens et al., 2018), using the effect size found in Baron and Hershey (1988), *d* = 0.21, as the lower and higher bound, though we note that the switch from a within-participants to a between-participants design makes it difficult to compare the effects. These were our chosen bounds because we want to not only look at whether there is evidence of an effect in our replication but also whether the effect size is affected by changes to the original study’s design (i.e., by switching to a between-participants design) and to the participant sample size and demographics. We found support for a larger effect in the replication (*t*(541.53) = 12.26, *p* < .001).

Next, we conducted a Welch’s two samples *t*-test for only the participants who indicated that they either definitely, or probably, should not consider the outcome when evaluating decision quality. This last analysis was conducted to attempt to replicate the finding from the original study that participants who acknowledged that they should not consider the outcome also show an outcome bias. As we measured outcome importance on a 5-point scale, participants who reported a response of 1 or 2 on this scale were considered as reporting that outcome should not be considered when evaluating a decision. Out of the 705 participants, 44 participants recorded an outcome importance value less than 3. We found that people who self-reported that they should not consider the outcome did in fact show an outcome bias (*t*(38.64) = 2.23, *M*_diff_ = 0.75, 95% CI [0.21, 1.08], *p* = .03, Cohen’s *d* = 0.64). Participants in the outcome success condition (*M* = 1.71, *SD* = 0.61) evaluated the decisions as higher than participants in the failure (*M* = 0.96, *SD* = 1.55) condition, even though participants in both groups indicated to some degree that they should not consider the outcome information.

We used the LeBel et al. (2019) paradigm for comparison of original and replication only with reference to signal and direction, yet with no reference to confidence interval overlap. This is because we switched the design from within-participants to between-participants, which makes such comparisons problematic.

In addition, the effect and CIs for the aggregate were computed using the *t*-values provided in the target article (given that no means and standard deviations were provided for the aggregate), whereas the effects for patient and physician were calculated from means and standard deviations (given that no *t*-values were provided). We note caution in comparing the two, given the many available methods to calculate effects for paired samples.

### Extensions

In a series of analyses, we examined the effect of outcome type (success vs. failure) and decision-maker type (physician vs. patient) on perceived outcome importance, perceived responsibility, and perceived norms.

#### Perceived Outcome Importance

**Figure 2:**
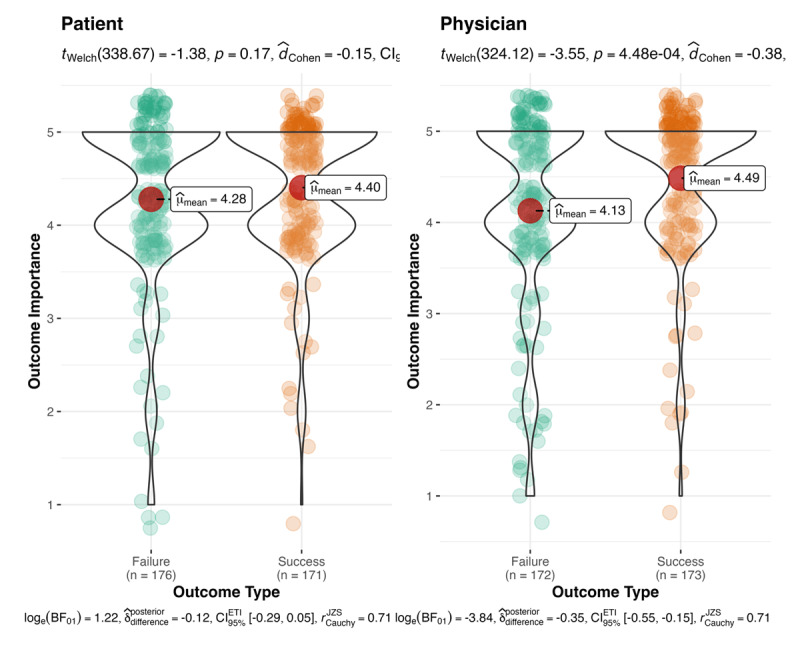
Outcome importance: decision-maker and outcomes. **Note:** The effect of outcome type (success vs. failure) and decision-maker type (physician vs. patient) on the perception of outcome importance.

First, we conducted a 2 outcome type (success vs. failure) × 2 decision-maker type (physician vs. patient) between-participants ANOVA to test whether these factors influenced the consideration of outcome importance on decision quality. We found that outcome type influenced outcome importance for evaluation of the decision (*F*(1, 688) = 12.64, *M*_diff_ = 0.23, *p* < .001, Cohen’s *f* = .14, 95% CI [–0.01, –0.27]; with Bonferroni corrections for multiple comparisons, *p* = .001). Successes (*M* = 4.44, *SD* = 0.80) were viewed as more important to consider when evaluating the outcome than failures (*M* = 4.20 *SD* = 0.98). We did not find support for the interaction (see Figure 2). Follow-up post hoc Welch’s *t*-tests suggest that participants in the physician condition (*t*(324.12) = 3.55, *M*_diff_ = 0.36, *p* < .001, Cohen’s *d* = 0.38, 95% CI [0.23, 0.53]) considered success as more important when evaluating the importance of outcome in a decision, but with no support for the effect for participants in the patient condition (*t*(338.67) = 1.38, *M*_diff_ = 0.13, *p* = .17, Cohen’s *d* = 0.11, 95% CI [–0.04, 0.26]).

#### Perceived Responsibility

**Figure 3:**
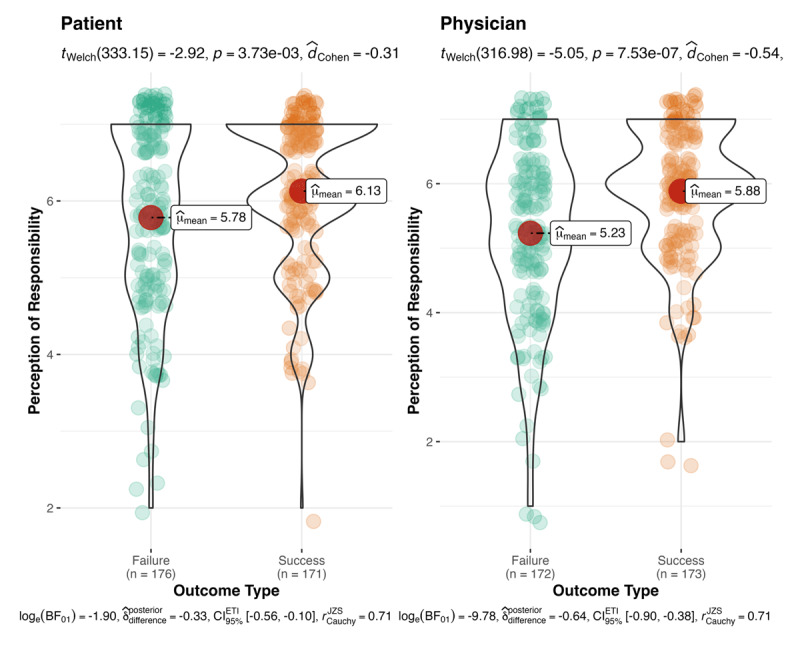
Perception of responsibility: decision-maker and outcomes. **Note:** The effect of outcome type (success vs. failure) and decision-maker type (physician vs. patient) on the perception of responsibility. When the outcome was a success, there were stronger perceptions that the decision-maker was responsible than when outcome was a failure.

Next, we conducted a 2 outcome type (success vs. failure) × 2 decision-maker type (physician vs. patient) between-participants ANOVA on perceived responsibility. We found that outcome type affected perceived responsibility (*F*(1, 688) = 32.36, *M*_diff_ = 0.49, *p* < .001, Cohen’s *f* = .22, 95% CI [0.07, 0.37]; with Bonferroni correction for multiple comparisons, *p* < .001). Participants assigned more responsibility to successful outcomes (*n* = 344, *M* = 6.01, *SD* = 1.0) compared to failures (*n* = 348, *M* = 5.51, *SD* = 1.31; see Figure 2). In addition, we found a main effect of decision-maker type on perceived responsibility (*F*(1, 688) = 20.32, *M*_diff_ = 0.39, *p <* .001, Cohen’s *f* = .17, 95% CI [0.02, 0.32]; with Bonferroni correction for multiple comparisons, *p* < .001). Patients (*n* = 347, *M* = 5.95, *SD* = 1.11) were perceived to be more responsible than physicians (*n* = 345, *M* = 5.56, *SD* = 1.24; see Figure 3). We found no support for an interaction.

### Exploratory Results (Not Pre-Registered)

#### Perceived Norms

**Figure 4:**
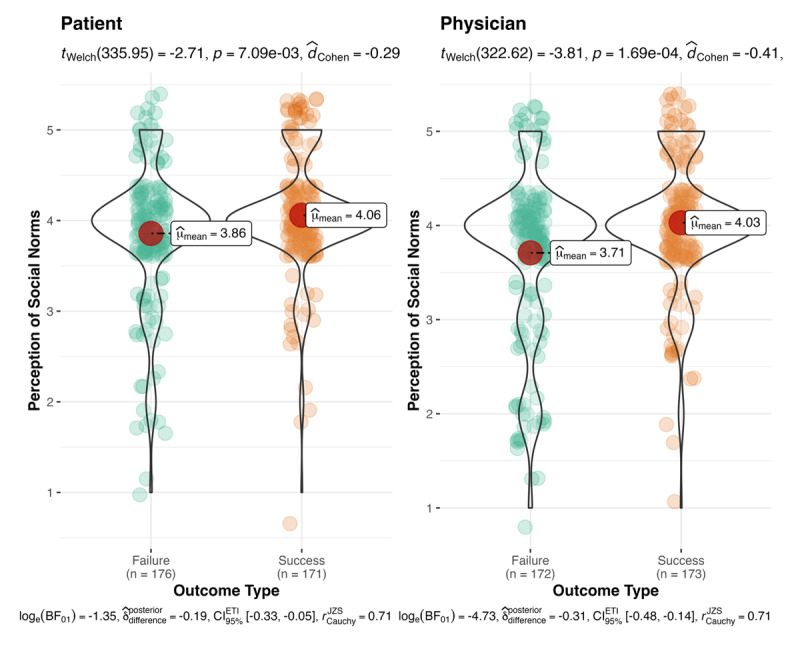
Perceived social norms: decision-maker and outcomes. **Note:** The effect of outcome type (success vs. failure) and decision-maker type (physician vs. patient) on the perceptions of social norms—whether others would perform the same action. When the outcome was a success, there were stronger perceptions others would perform the same action as the decision-maker than when outcome was a failure.

Lastly, we conducted a 2 outcome type (success vs. failure) × 2 decision-maker type (physician vs. patient) between-participants ANOVA on perceived norms. We found a main effect for outcome type (*F*(1, 688) = 21.54, *M*_diff_ = 0.25, *p* < .001, Cohen’s *f* = .18, 95% CI [0.03, 0.33]; with Bonferroni correction for multiple comparisons, *p* < .001). Successful outcomes (*n* = 344, *M* = 4.04, *SD* = 0.65) were perceived more normal than failed outcomes (*n* = 348, *M* = 3.78, *SD* = 0.82; see Figure 4). We found no support for a main effect of decision-maker (*F*(1, 688) = 2.43, *M*_diff_ = 0.09, *p* = .12, Cohen’s *f* = .06, 95% CI [–0.09, 0.21]). Decisions made by patients were perceived as being more normative (*n* = 347, *M* = 3.96, *SD* = 0.70) than those made by physicians (*n* = 345, *M* = 3.87, *SD* = 0.79). We found no support for an interaction.

#### Mediation Analyses

In the Mediation Analyses section of the Supplemental Materials, we report exploratory mediation analyses, conducted using the mediation package in R (Tingley et al., 2014), to examine the possibility that outcome importance, responsibility, or norms at least partially explain the effect of outcome type on evaluations of the decision (i.e., accounted for outcome bias). As these are exploratory, we caution against over-interpreting the results, which only indicated an indirect effect of norms on the relationship between outcome and decision evaluation.

## Discussion

We conducted a well-powered, pre-registered direct replication and extension of Baron and Hershey’s (1988) findings on outcome bias using a between-participants design. We concluded a successful replication with signal and direction consistent with that of the target article’s findings. Outcomes affected perceived decision quality, in that a decision resulting in positive outcomes was judged as better than a decision resulting in negative outcomes. Moreover, we extended the work by exploring perceived outcome importance, responsibility, and norms as potential mechanisms of outcome bias.

In this replication, we focused on a single scenario focused on medical decision-making with various outcomes, limiting the generalizability further from the original 15-scenario design (for a discussion of the issue, see Yarkoni, 2022). The switch to this single scenario could have contributed to the larger effect size. Replications are meant to reproduce and re-examine studies. We contributed with a minor extension of the original’s study design by adapting from within-participants to between-participants, yet this should only be taken as a first step for future tests of the generalizability of this paradigm. We considered it important to first revisit and re-examine the replicability of one of the most impactful classics regarding outcome bias.

### Successful Replication of Outcome Bias

We found support for outcome bias and with much larger effects (patient: original *d* = 0.21, replication *d* = 0.77, 95% CI [0.62, 0.93]; physician: original *d* = 0.53, replication *d* = 1.1, 95% CI [0.94, 1.26]). We also found that participants who acknowledged that they should not consider outcome information when evaluating the decision still showed outcome bias.

Overall, the present study can be considered a successful replication attempt. Though comparing effect sizes of within-participants and between-participants designs should be done with caution, some studies in judgment and decision-making that examined attributions (e.g., omission bias) reported larger effect sizes for within-participants designs than between-participants designs. In this case, we observe comparable or stronger effects in our replication using a between-participants design compared to the target’s within-participants design. It is possible that in a within-participants design, participants anchor their evaluation for one outcome type when providing a subsequent decision evaluation or that the scenarios we chose were the most impactful. In neither the original study nor this replication was there a baseline condition (i.e., where outcome is not provided) that would allow us to determine how a mere presence of outcome, positive or negative, affects decision evaluation. However, providing evidence that outcome bias holds even with a different experimental design indicates a broad phenomenon that affects how individuals view others’ decisions, even when they are aware that outcomes should not be considered when evaluating decisions.

### Extensions

#### Perceived Outcome Importance

Outcomes were considered more important for assessment of decision quality when they were positive compared to negative. Practically, this finding is important because the outcome does not directly relate to the decision quality as Baron and Hershey (1988) suggest. People may take the outcome into account more when the decision leads to a success, and thus they may be more optimistic about the decision quality compared to when the decision leads to a failure. However, we did not find support for the hypothesis that outcome importance related to the evaluation of the decision or if it could at least partially explain outcome bias. Sezer et al. (2016) found that making the intentions of others’ decisions more salient before asking individuals to evaluate these decisions, especially for decisions pertaining to multiple joint decision-makers, aided the mitigation of outcome bias. This suggests that individuals do not naturally introspect about how outcomes are integrated into a decision evaluation, but they can be prompted to do so when asked to make a more effortful consideration of a decision’s rationale.

The fact that both approaches decreased outcome bias could indicate that individuals work from an implicit assumption that outcomes are a result of decisions whereby those decisions’ thought processes are not known. When asked to evaluate decisions, individuals default to evaluating the outcome and the decision-maker’s contribution to that outcome. Making salient the decision-maker’s rationale or the lack of agency in the outcome prompts individuals to reconsider that contribution to the outcome.

#### Perceived Responsibility

Successful outcomes were perceived as more accountable than failures. As such, successful decisions are perceived to be caused by the decision-maker to a greater degree than decisions that end in failure. This is somewhat surprising, as there are compelling accounts for the opposite to be true. In their study, Tostain and Lebreuilly (2008) had participants read an account of an unintentional road accident with either mild or severe outcomes for those involved in the accident. Participants judged the driver as having more responsibility in the severe outcome condition than in the mild outcome condition. One may have expected higher responsibility for unsuccessful outcomes, given that regret has been shown to be higher for actions compared to inactions that lead to failures (Feldman & Albarracín, 2017; Kahneman & Tversky, 1982). Hence, an observation of a decision leading to a failure would lead to a vicarious perception of such regret.

Our results also suggest that patients are perceived to be more responsible for the outcome of the decisions compared to physicians. This may be because people who judge decisions tend to assign more responsibility to the people whom the decision will be affecting (i.e., the patient) rather than the person who will be carrying out the execution of the decision (i.e., the physician). It would be interesting to investigate whether these differences in perceptions around responsibility extend to other domains, as the patient-physician dynamic may have domain-specific connotations for individuals. These findings have some practical implications for how decisions are judged and when we may be biased to be optimistic or pessimistic regarding the correctness of a decision. However, we did not find support for the hypothesis that responsibility related to the evaluation of the decision or whether it could at least partially explain outcome bias.

#### Perceived Norms

Decisions resulting in successful outcomes were perceived more normative than those leading to failed outcomes. This suggests that outcome bias may be the result of participants believing that the outcome was foreseeable by others who would have made the correct choice, like the participants in the success condition or unlike the participants in the failure condition. We explored whether this perception could help explain outcome bias. The results showed that it explained approximately 15% of the variance in the outcome bias. As such, outcome bias can be partially explained by the perception of what others would have done given the same decision. This warrants future work to look at whether the social norms around outcomes can be disassociated from the perceptions of decisions, as well as gaining a better understanding of why these norms may be in place. As this was an analysis that extended the original paper, this result requires further replication, as it has implications on whether the presence of outcome bias is based on social knowledge or on a true belief on the part of the individual. This result is only a very early indication of the role of norms in outcome bias and hence requires further investigation.

#### Broader Importance of Outcome Bias

The medical decisions outcome bias scenario that we aimed to replicate from the target received follow-up research extending the phenomenon beyond the lab and examining moderating factors. For example, it has been suggested that outcome bias is weakened by seniority (Liaw et al., 2019) and strengthened by salience of professional identity (Fan et al., 2021). Medical decisions involve a unique information asymmetry on the part of patients, who have less expertise and experience and are less familiar with the base rates used to inform their decisions (Bar-Hillel, 1980). This makes the domain well suited to investigating outcome bias.

If outcome bias is a broad generalizable phenomenon that impacts decision-making and evaluations, then it may have broad implications for many domains.

In social psychology, outcomes were shown to positively influence perceptions of a salient in-group but did not affect these perceptions when they were detrimental to the in-group (Mackie & Ahn, 1998). In economic decision-making, outcomes were shown to affect trust in financial agents, even if the outcomes of risky investments in these agents were randomly determined (König-Kersting et al., 2021). There has even been evidence that outcome bias drives changes in strategy among basketball coaches, who were shown to be more likely to revise strategies after a loss even if the losses were narrow and uninformative (Lefgren, Platt & Price, 2015). Similarly, outcome bias has also been explored in the evaluation of players who participate in penalty shootouts in football (Kausel et al., 2019). Future replications may aim to revisit these findings to further demonstrate the generalizability and importance of outcome bias with potential interventions aimed to mitigate it.

There are also potential implications for the research life cycle, peer review, and publication in academic journals (Callaham et al., 1998). For example, Emerson et al. (2010) provided two sets of forged manuscripts to peer reviewers. The manuscripts were identical, except for the results—positive results or null (no support found). A large proportion of peer reviewers recommended the study with the positive results to be published, compared with the study that had null results. Similar results were also found in Callaham et al. (1998). The study showed that one of the best predictors for research abstracts to be accepted for presentation by journals was having ‘positive results’ in the form of statistically significant results and strong effect sizes (Callaham et al., 1998). These examples exemplify that outcome bias may exist in academia, which may indirectly result in the conscious or subconscious use of practices aimed at increasing chances of ‘positive results’. Evaluations of research quality based on outcomes may result in strong file drawer or publication bias in the literature. Insightful results may be rejected for publication because the results were not ‘positive’, and the research done that led to these results may possibly be perceived as inferior. This is where solutions such as Registered Reports and outcome blinding allow for researchers and reviewers to disassociate the outcome of research results from evaluations of their merit.

#### Constraints on Generality

We note the limited generalizability of our replication given that the experimental design is based on a single scenario. We outlined other work that followed on outcome bias since to illustrate the potential impact of outcome bias, yet we caution against generalizing findings from this replication to other contexts without further work. We aimed to broaden the understanding of the phenomenon, extending the target article’s study by switching to a more naturalistic between-participants and replicating overall findings with a more heterogenous population with added extensions, yet we see much potential for more follow-up research to revisit follow-up research and further examine its generalizability to other contexts and designs.

#### Limitations and Future Directions

A limitation is that the present study only examined conditions 1–4 of the original study’s 15 total conditions. Conditions 5–8 applied the same scenarios to liver surgery rather than heart surgery, while conditions 9–15 presented scenarios where positive or negative tests were conducted to detect certain diseases. We had chosen to focus on conditions 1–4, as this made the replication easier to administer between-participants and allowed us to extend the results from these conditions in more detail. Given that these different conditions also produced evidence of outcome bias (and were negative mean values of decision evaluation in conditions 9–15 even when the outcome was successful), conditions 1–4 were chosen as the focus. Using the choice of whether to have surgery is also likely to be easier and more relatable for a layperson to understand than testing for diseases. We discussed possible implications of the target article’s and our replication’s findings, yet we caution against over-interpretation given that the investigations were focused on a single domain in a specific context. Future replication studies could aim to replicate the other conditions to extend this work to the other types of scenarios. Given that the target study has served as the foundations for investigating outcome bias, and our replication study aimed at revisiting these foundations, we can now at least adjust our estimates to consider outcome bias as a more solid stable phenomenon that replicates 35 years later, with a between-participants design and with a more diverse sample, to inform future work.

We note that one potential explanation for the strong effects we observed in the replication compared to the original study may be a result of the adjusted design and adding comprehension checks. It is possible that by making certain information with the scenario more salient (including the scenario outcome specifically) in the comprehension check questions, participants tended to focus their attention on that information in their decision evaluations, and there is also the possibility that they might use that as a cue to try and answer in a manner that they thought would be expected of them, taking decision outcome into account. We included these comprehension checks to ensure that participants were attentive to the details of the scenario, and the between-participants design limited their ability to deduce experimenters’ intent and purpose of study. Future work could address this by checking awareness of the stimuli by other means, such as randomizing whether the comprehension checks are shown before or after participants provide their responses.

Participants were prone to an outcome bias even when they reported that outcomes are not important when evaluating the quality of a decision, with only a small subset of participants indicating they think outcomes are important (*n* = 44, 6.24% of all participants). This finding is particularly interesting in hinting at the possibility of outcome bias being unconscious. However, the sample size for this analysis was fairly small. It would be useful for future studies to look into this aspect of outcome bias further and investigate whether it is indeed an unconscious bias.

Participants mentioned ethical issues when evaluating the decisions. When asked to justify their evaluations of decisions, 6.8% of the participants reported that they gave a poor rating due to ethical concerns. A majority of these concerns came from the conditions in which the physician was making the decision and not the patient. Participants in these conditions believed that the patient should have the right to decide whether to carry out the operation or not. Future studies examining the effect of outcome bias could replicate Experiments 3, 4, and 5 of Baron and Hershey (1988) to extend the present replication beyond decision scenarios that involve medical ethics. Related literature has already extended the findings of outcome bias to other domains, such as judgments of ethicality, trustworthiness, and financial allocation, and thus future work can seek to understand whether there are contexts where outcome bias can be mitigated. While we observed outcome bias regardless of the decision-maker, it could well be that perceptions of agency have an effect on the extent of outcome bias. It is possible that participants have differing views of outcomes depending on personal or societal principles of autonomy, which can be an avenue for future work.

## Additional Files

The additional files for this article can be found as follows:

10.5334/irsp.751.s1 Supplemental file. Open Science Disclosures, Effect Size Calculations, Materials used in Experiment, Mediation Analyses, and Analysis using all Data.

10.5334/irsp.751.s2 Review file 1. Open Review Response for the 1st round of review, including Response to Editor and Response to Reviewer.

10.5334/irsp.751.s3 Review file 2. Open Review Response for the 2nd round of review, including Response to Editor and Response to Reviewer.

## Figures and Tables

**Table:** 


ROLE	SRIRAJ AIYER	HOI CHING KAM AND KA YUK NG	NATHANIEL A. YOUNG	JIAXIN SHI	GILAD FELDMAN

Conceptualization					X

Pre-registrations		X			X

Data curation					

Formal analysis	X	X	X		

Funding acquisition					X

Investigation		X			X

Methodology		X			X

Pre-registration peer review/verification		X		X	X

Data analysis peer review/verification	X	X	X		

Project administration					X

Resources					X

Supervision				X	X

Validation	X		X		

Visualization	X		X		

Writing—original draft		X	X		

Writing—review and editing	X				X


## Citation of the Target Research Article

Baron, J., & Hershey, J. C. (1988). Outcome bias in decision evaluation. *Journal of Personality and Social Psychology*, *54*(4), 569. https://www.sas.upenn.edu/~baron/papers/outcomebias.pdf.

## Author Information

Sriraj Aiyer is a research assistant with the University of Oxford’s Department of Experimental Psychology. His research focuses on confidence and trust in a metacognition lab.

Hoi Ching Kam and Ka Yuk Ng were students at the University of Hong Kong during the academic year 2018–9.

Nathaniel A. Young was a PhD student with the psychology department at DePaul University. His research focuses on emotion and decision-making.

Jiaxin Shi was the teaching assistant at the University of Hong Kong psychology department during the academic year 2018–9.

Gilad Feldman is an assistant professor with the University of Hong Kong psychology department. His research focuses on judgment and decision-making.
