# Supplementary material for: Outcomes Affect Evaluations of Decision Quality: Replication and Extensions of Baron and Hershey’s (1988) Outcome Bias Experiment 1
Source: Int Rev Soc Psychol. 2023 Jul 28;36:12. doi: 10.5334/irsp.751 (PMC12372742; doi:10.5334/irsp.751)
Supplement: Supplemental file. — Open Science Disclosures, Effect Size Calculations, Materials used in Experiment, Mediation Analyses, and Analysis using all Data. [file irsp-36-751-s1.pdf]

Outcomes affect evaluations of decision quality: Replication and extensions of Baron and Hershey's (1988) Outcome Bias Experiment 1

Supplemental Materials

Open Science Disclosures 1
Data collection 1
Conditions reporting 1
Variables reporting 2
Original article: Effect size calculations for 3
Effect size calculations and power analysis 3
Condition 1 vs. Condition 2 – Decision maker: Physician; Outcome of operation: Success vs. Failure. 3
Condition 3 vs. Condition 4 – Decision maker: Patient; Outcome of operation: Success vs. Failure. 3
Sensitivity analysis on the collected sample of 692 4
Materials used in the experiment 5
Mediation Analyses 6
Mediation analysis discussion 8
Analysis Using all Data (Including Data Exclusions) 10
Replication: Decision quality 10
Comparing replication to original: Equivalence 11
Extensions 12
Perceived outcome importance. 12
Perceived Responsibility. 13
Perceived Norms. 13

Open Science Disclosures

Data collection

Data collection was completed before analyzing the data.

Conditions reporting

All collected conditions are reported.

Variables reporting

All variables collected for this study are reported and included in the provided data.

Original article: Effect size calculations for

Effect size calculations and power analysis

Please see accompanying Rmarkdown and output added to the OSF folder.

Descriptives and statistics used from the target article:

Decision maker: Physician; Success vs. Failure (Conditions 1 vs. 2).

Condition 1: Mean = 0.85; SD = 1.62; N = 20
Condition 2: Mean = -0.05; SD = 1.77; N = 20

Decision maker: Patient; Success vs. Failure (Conditions 3 vs. 4)

Condition 3: Mean = 1.00; SD = 1.05; N = 20
Condition 4: Mean = 0.75; SD = 1.26; N = 20

Sensitivity analysis on the collected sample of 692

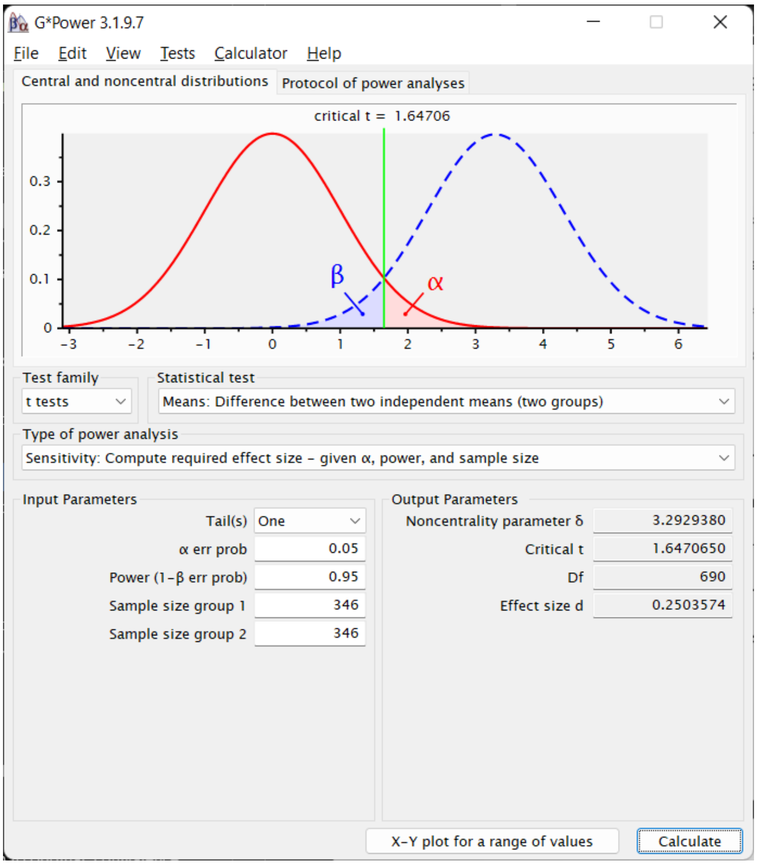

t tests – Means: Difference between two independent means (two groups)

**Analysis:**	Sensitivity: Compute required effect size		
**Input:**	Tail(s)	=	One
	α err prob	=	0.05
	Power (1−β err prob)	=	0.95
	Sample size group 1	=	346
	Sample size group 2	=	346
**Output:**	Noncentrality parameter δ	=	3.2929380
	Critical t	=	1.6470650
	Df	=	690

Materials used in the experiment

See provided Qualtrics in the OSF folder.

Correlation Between Items

Below is a visualisation showing the correlation between the four dependent variables (Decision Evaluation, Outcome Importance, Ratings of Responsibility, Ratings of Norms across all participants. The bottom-left triangle of panels show plots of the two variables that “intersect” at that square against each other. The top-right triangle show correlation coefficient values via Pearson’s Correlation. On the diagonals are the variables for that row and column, as well as a histogram of the distribution of values for that variable. The correlations values with a single asterisk are statistically significant ($p < .05$), whilst values with three asterisks have p values less than .001.

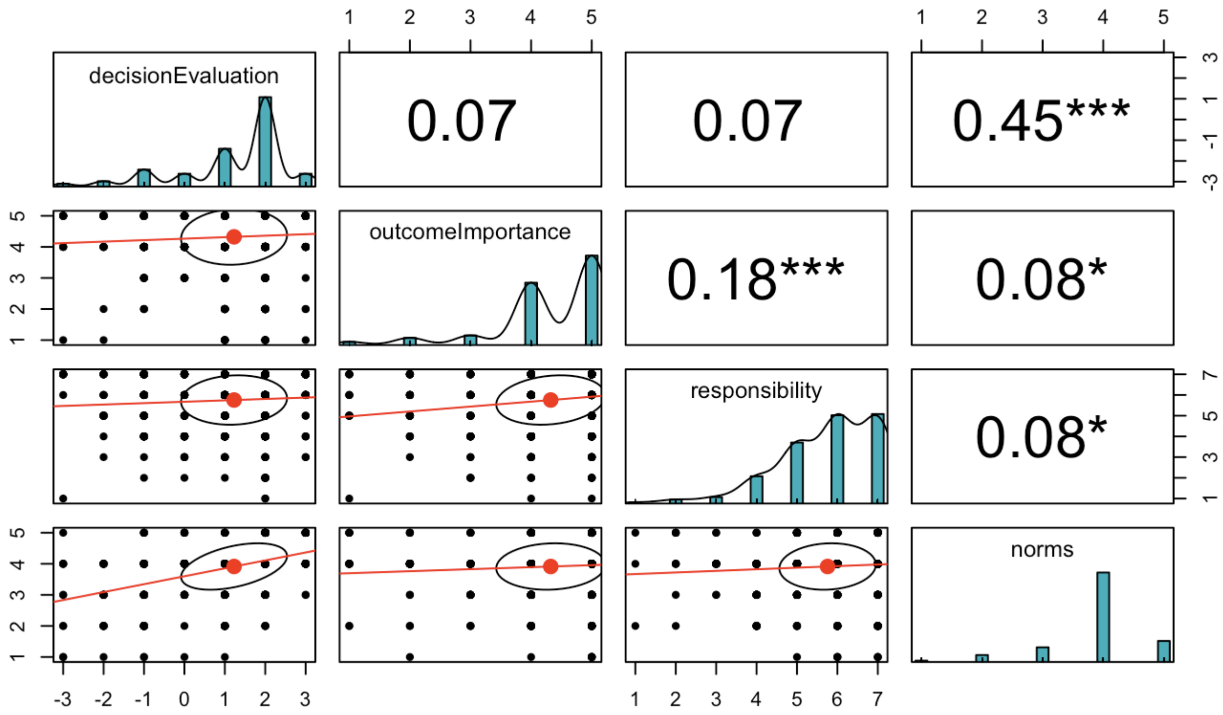

Mediation Analyses

We conducted a mediation analysis using the mediation R package (Tingley et al, 2014) to explore if perceived outcome importance, responsibility, or norms would account for outcome bias. It is worth noting these mediation analyses were not mentioned in our preregistration. First, the outcome type (success vs failure) was regressed on each possible mediator. Then each mediator and the outcome type was regressed on the outcome of the decision evaluation. Lastly, each estimate of the causal mediation effect (indirect effect: IE) was computed for each of 5,000 bootstrapped samples, and the 95% confidence interval was computed by determine the indirect effect at the 2.5% and 97.5% percentiles for the mediator.

The effect of outcome (success vs failure) was not found to be mediated by outcome importance. As figures 5, 6 and 7 illustrate, the regression coefficient between outcome and decision evaluation was significant. The bootstrapped indirect effect of outcome importance was .008, 95% CI [-.02, .04]. The indirect effect was hence found to be statistically insignificant ($p = .50$). The indirect effect of responsibility was .002, 95% CI [-.04, .04], with $p = .88$, indicating statistical insignificance. However, the indirect effect of norms was .18, 95% CI [.09, .26], $p < .001$. Hence, we found the indirect effect of norms on the relationship between outcome and decision evaluation to be statistically significant.

Figure 1

Outcome importance: Mediation analysis on the relationship between outcome and decision evaluation.

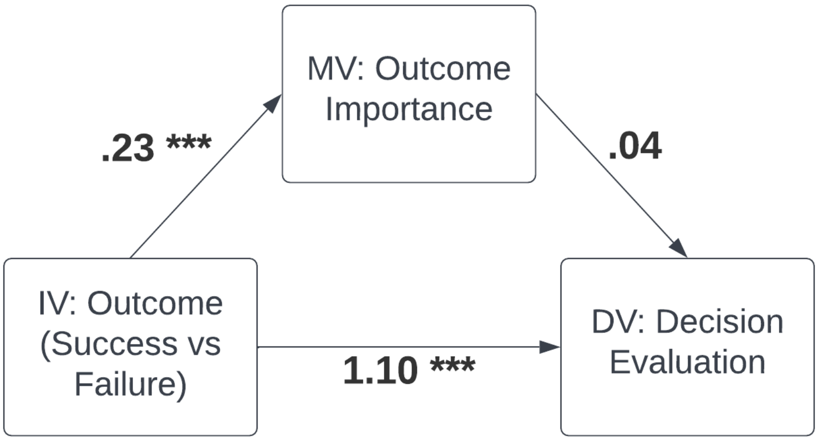

Note. *** denotes $p < .001$.

Figure 2

Responsibility: Mediation analysis on the relationship between outcome and decision evaluation.

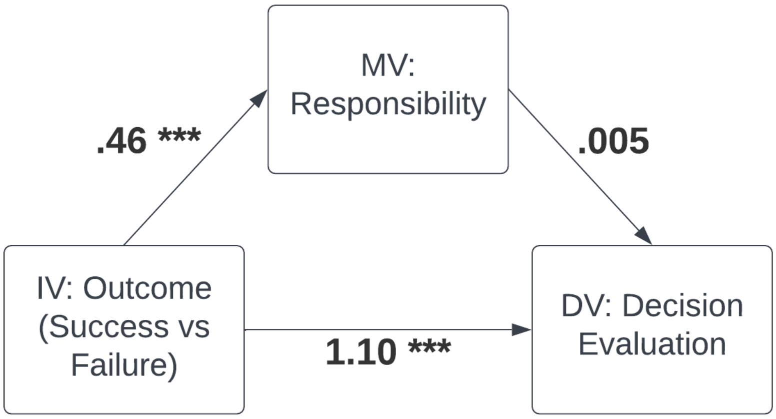

Note. *** denotes $p < .001$.

Figure 3

Norms: Mediation analysis on the relationship between outcome and decision evaluation.

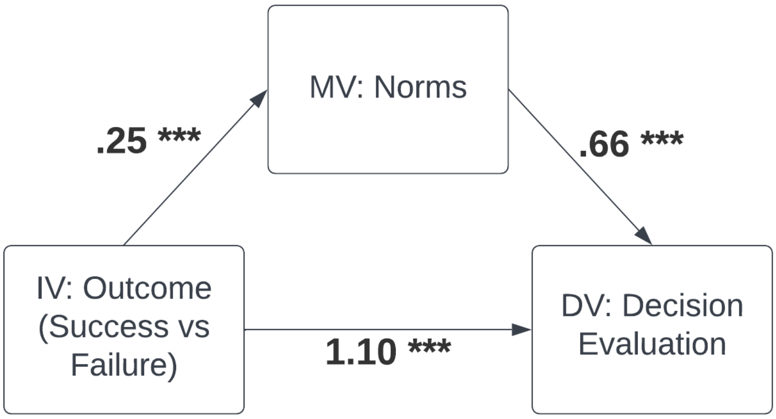

Mediation analysis discussion

We also conducted mediation analyses to see whether outcome importance, perceived responsibility and perceived norms mediated the relationship between outcome and decision evaluation (see Supplemental Materials). We observed some evidence that outcome bias may be driven by a normative account of decision evaluation, in that we expect others to also consider outcomes when evaluating decisions. We should note that this is fairly weak evidence and hence would require future work in order to better explore the mechanisms that drive outcome bias. This normative account would also conflict with the unconscious account of outcome bias presented previously, and thus requires further investigation.

Table 1

Mediation Analysis.

Test	DV	IV	□	*p*	Effect size (Cohen's *d*)	95% CI
	Outcome importance	Outcome type	.23	< .001	.26	.11, .41
	Evaluation	Outcome type	1.10	< .001	.92	.77, 1.08
	Evaluation	Outcome importance	.04	= .47	.03	-.06, .13
IE	Evaluation	Outcome type via Outcome importance	.008	= .50		-.02, .04
Prop. Mediated			.008	= .50		-.02, .03
	Responsibility	Outcome type	.46	< .001	.39	.24, .54
	Evaluation	Outcome type	1.10	< .001	.92	.77, 1.08
	Evaluation	Responsibility	.004	= .90	.004	-.06, .07
IE	Evaluation	Outcome type via Responsibility	.002	= .88		-.04, .04
Prop. Mediated			.002	= .88		-.04, .04
	Similar action	Outcome type	.25	< .001	.34	.19, .49
	Evaluation	Outcome type	1.10	< .001	.92	.77, 1.08
	Evaluation	Similar action	.66	< .001	.62	.55, .77
IE	Evaluation	Outcome type via Similar action	.17	< .001		.09, .26
Prop. Mediated			.15	< .001		.09, .23

Note. IE represents indirect effect. Prop Mediated represents the proportion of the variance that the mediator can explain between outcome type and the evaluation of the decision.

Analysis Using all Data (Including Data Exclusions)

Replication: Decision quality

We conducted three analyses to examine the replication hypotheses. First, to test outcome bias, we ran a 2 outcome type (success / failure) x 2 decision maker (physician / patient) between subjects ANOVA. We found support for a main effect of decision maker over perceived decision quality ($F(1, 701) = 4.82$, $M_{diff} = .195$, $p = .028$, Cohen's $f = .08$, 90% CI [.02, .14). Patients' decisions were evaluated as higher quality (N = 353, $M = 1.33$, $SD = 1.19$) than that of physicians' (N = 352, $M = 1.13$, $SD = 1.42$).

Furthermore, we found support for a main effect of outcome type . ($F(1, 701) = 154.2$, $M_{diff} = 1.10$, $p < .001$, Cohen's $f = .47$, 90% CI [.40, .53]). Decisions resulting in positive outcomes were evaluated as higher quality (N = 353, $M = 1.78$, $SD = .81$) compared to those resulting in negative outcomes (N = 352, $M = .68$, $SD = 1.47$).

We conclude that these findings as a successful replication of the original experiment.

We further tested and found an interaction of outcome type and decision maker type ($F(1,701) = 9.19$, $p = .003$, Cohen's $f = .11$, 90% CI [.05, .18]). Follow-up post-hoc t-tests suggest that physicians ($t(266.75) = 10.34$, $M_{diff} = 1.37$, $p < .001$, Cohen's $d = 1.11$, 95% CI [.94, 1.26],) and patients ($t(282.90) = 7.05$, $M_{diff} = -.83$, $p < .001$, Cohen's $d = .75$, 95% CI [.59, .90]) were evaluated as more correct when they resulted in success than when they resulted in failure confirming an outcome bias effect for both the physician and patient conditions. However, physicians' decisions were evaluated as lower quality than patients' decisions ($t(343.39) = -3.02$, $M_{diff} = .47$, $p = .003$, Cohen's $d = -.32$, 95% CI [-.47, -.17]) when they resulted in failure, but physicians and patients were evaluated as similarly correct

when they resulted in success (t(350.16) = .80, M_{diff} = -.07, p = .42, Cohen's d = .09, 95% CI [-.06, .23]).

Comparing replication to original: Equivalence

We conducted a follow-up equivalence test to determine if the outcome bias effect found in the replication was equivalent to the effect found in Baron and Hershey (1988). We used two one-sided tests TOST procedure (Lakens et al., 2018), using the effect size found in Baron and Hershey (1988), d = .21 as the lower and higher bound. We found that the replication's effect size is non-inferior / superior to the original (Test to Reject Null Significance Hypothesis: t(546.11) = 12.29, p < .001), Mean Difference = 1.10, 90% CI [0.96, 1.25], Hedges' g = 0.93, 90% CI = [0.80, 1.06]. This suggests that the outcome bias effect found in the current study is not equivalent, but importantly that it is significantly stronger than the one found in the original experiment. Thus, given the power of the present study, compared to the original experiment, the outcome bias effect may be stronger than originally estimated based upon the original Baron and Hershey (1988) experiment.

Next, a t-test was conducted for only the participants who indicated that they either definitely, or probably, should not consider the outcome when evaluating decision quality. This last analysis was conducted to attempt to replicate the finding from the original study that participants who acknowledged that they should not consider the outcome also show an outcome bias. As we measured outcome importance on a 5-point scale, participants who reported a response of 1 or 2 on this scale were considered as reporting that outcome should not be considered when evaluating a decision, Out of the 705 participants, 44 participants recorded an outcome importance value less than 3. We found that people who self-reported that they should not consider the outcome did in fact show an outcome bias ($t(39.68) = 2.36$, $M_{diff} = .80$, 95% CI [.12, .1.50], $p = .023$, Cohen's $d = .66$). Participants in the outcome success condition ($M = 1.67$, $SD = .62$) evaluated the decisions as higher than participants in the failure ($M = .86$, $SD = 1.62$) condition, even though participants in both groups indicated to some degree that they should not consider the outcome information.

Extensions

In a series of analyses we examined the effect of outcome type (success vs failure) and decision maker type (physician vs patient) on perceived outcome importance, perceived responsibility, and perceived norms. In addition, mediation analyses were conducted to explore the possibility that outcome importance, responsibility, or norms at least partially explain the effect of outcome type on evaluations of the decision (i.e. accounted for outcome bias).

Perceived outcome importance.

First, we conducted a 2 outcome type (success vs failure) x 2 decision maker type (physician vs patient) between subjects ANOVA to test if these factors influenced the consideration of outcome importance on decision quality. We found that outcome type

influenced outcome importance for evaluation of the decision (*F*(1, 701) = 11.71, M_{diff} = -.23, *p* < .001, Cohen's *f* = .13, 95% CI [-.01, -.27],). Successes (*M* = 4.42, *SD* = .81) are viewed as more important to consider when evaluating the outcome than failures (*M* = 4.19 *SD* = .98). We found no support for an interaction. Follow-up post-hoc t-tests suggest that participants in the physician condition (t(332.52) = 3.45, Mdiff = .35, p < .001, Cohen's d = .37, 95% CI [.22, .52]) considered success as more important when evaluating the importance of outcome in a decision, but this was not the case for participants in the patient condition (t(342.78) = 1.28, Mdiff = .12, p = .20, Cohen's d = .10, 95% CI [-.04, .25]).

<u>Perceived Responsibility.</u>

Next, we conducted a 2 outcome type (success vs failure) x 2 decision maker type (physician vs patient) between-subjects ANOVA on perceived responsibility. We found that outcome type affected perceived responsibility (*F*(1, 701) = 27.7, M_{diff} = .46, *p* < .001, Cohen's *f* = .20, 95% CI [.05, .35]). Participants assigned more responsibility to successful outcomes (N = 353, *M* = 5.97, *SD* = 1.05) compared to failures (N = 352, *M* = 5.50, *SD* = 1.32; see Figure 2). In addition, we found a main effect of decision maker type on perceived responsibility (*F*(1, 701) = 21.1, *Mdiff* = .41,. *p* < .001, Cohen's *f* = .17, 95% CI [.02, .32]). Patients (N = 353, *M* = 5.94, *SD* = 1.14) were perceived to be more responsible than physicians (N = 352, *M* = 5.53, *SD* = 1.26). We found no support for an interaction.

<u>Perceived Norms.</u>

Lastly, we conducted a 2 outcome type (success vs failure) x 2 decision maker type (physician vs patient) between-subjects ANOVA on perceived norms. We found a main effect for outcome type (*F*(1, 701) = 20.57, M_{diff} = .25, *p* < .001, Cohen's *f* = .17, 95% CI [.02, .32]). Successful outcomes (N = 353, *M* = 4.03, *SD* = .66) were perceived more normal than failed outcomes (N = 352, *M* = 3.78, *SD* = .82). The main effect of decision maker was not statistically significant (*F*(1, 701) = 3.18, M_{diff} = .09, *p* = .07, Cohen's *f* = .07, 95% CI [-

.08, .22]). Decisions made by patients were perceived as being more normative (N = 353, M = 3.95, SD = 0.70) than those made by physicians (N = 352, M = 3.86, SD = 0.80). We found no support for an interaction.
