# Supplementary material for: Outcomes Affect Evaluations of Decision Quality: Replication and Extensions of Baron and Hershey’s (1988) Outcome Bias Experiment 1
Source: Int Rev Soc Psychol. 2023 Jul 28;36:12. doi: 10.5334/irsp.751 (PMC12372742; doi:10.5334/irsp.751)
Supplement: Review file 1. — Open Review Response for the 1st round of review, including Response to Editor and Response to Reviewer. [file irsp-36-751-s2.pdf]

Reply to decision letter reviews:
Baron and Hershey's (1988) replication and extensions

We would like to thank the editor and the reviewers for their useful suggestions and below we provide a detailed response as well as a tally of all the changes that were made in the manuscript. For an easier overview of all the changes made, we also provide a summary of changes.

Please note that the editor's and reviewers' comments are in **bold** with our reply underneath in normal script.

A track-changes comparison of the previous submission and the revised submission can be found on: https://draftable.com/compare/GWTZEpSRaQRA

Summary of changes

Below we provide a table with a summary of the main changes to the manuscript and our response to the editor and reviewers:

**Section**	**Actions taken in the current manuscript**
General	We addressed comments on the original study's generalizability, aspects of reporting analyses and provided more details on methods (e.g. exclusion criteria, pre-registrations) among other small changes.
Introduction	The introduction is more concise, with more information provided on the replication closeness criteria, the reasoning behind the original study choice for replication (with its effect and sample sizes) and the use of the pre-registrations.
Methods	We added more details to the Methods on the exclusion criteria and survey flow, which should address comments about the participant sample.
Results	We added to Results by including corrections and clarity on which statistical tests are applied, as well as providing higher quality figures.
Discussion	The Discussion has been rewritten to address generalizability, impact on subsequent research, and address other potential limitations or rationales for the observed results.
Reporting	We sharpen the language around how effects are reported.
Supplementary materials	We added a correlational analysis to the Supplementals to look at measure independence. We also added references to the power analysis in the Supplementals.

Response to Editor: Prof. Hans IJzerman

> **First of all, my apologies in the delay in getting back to you. I had a really hard time getting reviewers for this manuscript in the first place (with many reviewers either not responding or rejecting) and one of the reviewers that I invited and who had accepted went unresponsive, no matter how I tried to contact him. I did invite the original first author, as I think it is important to get an original author's first take and I think I have enough experience in conducting replication research to contextualize the original author's feedback if they oppose replications. Unfortunately, he rejected as he felt that it was not necessary to conduct another replication, as sufficient conceptual replications have been conducted (note that the original author indicated not to want to be anonymous in the review process, so I feel comfortable sharing that this was his take). You can take that for what it's worth.**
>
> **I did receive one solid review from Ivan Ropovik; I am very grateful for his support in providing you with feedback. Given the already lengthy delay, I wanted to provide you with feedback on your manuscript. I asked him for advice on the methods, unfortunately, I was unable to get a content expert, but with such replications I feel sufficiently comfortable making these judgments myself. Before reading his advice and as is my custom, I read the manuscript independently.**

Thank you for the reviews obtained, your feedback, and the invitation to revise and resubmit.

Important: As noted in a follow-up email by the corresponding author, we would like address the need for disclosures. We noted the following in the manuscript in the Declaration of Conflict of Interest:

> Disclosure: Prof. Hans Rocha IJzerman, the handling editor of this manuscript in the International Review of Social Psychology, has collaborated and coauthored with the corresponding author on several large scale open-science replication collaborations. The corresponding author is one of the many scholars who signed up to participate in the handling editor's coordinated projects of STRAEQ-2 and CREP Africa.
>
> To address any potential concerns, the corresponding author has requested to make all peer review regarding this manuscript publicly available. The handling editor and the journal have agreed to switch to an open peer review track.

> **You will see that this independent reading introduced some divergence and some convergence. I am personally more critical about the pre-registration and the writing and I will ask you to follow points that I have in my criticism.**
>
> **One point of convergence that Ropovik and I had was on the strength of the original study. Ropovik suggested you resolve this in the discussion (see his last comment). I really struggled with what decision to take. Let's face it, the original study was badly designed. In a modern study, one shouldn't accept studies that rely on single scenarios. I personally would not run a replication study like this anymore, but I would seek to improve the design. I would include the original scenario, but then write additional scenarios to increase the generalizability of the study. I am willing to step away from this point for your manuscript, given the impact of the original, provided that you write a really strong paragraph in your discussion outlining the problems of the original study's design as it pertains to generalizability. You can look at the Judd et al (2012) article and/or the Yarkoni article that Ropovik cites. Without such a paragraph, I am not able to accept this manuscript.**

Thank you for the feedback.

We added an extra paragraph to the start of the Discussion section. We hope that including this paragraph preemptively tones down the remainder of the Discussion such that we do not encourage overgeneralizations. To summarize here, our view is that replications should be focused on the task of repeating original studies and examining their reproducibility and replicability of findings, and so we see it is to be outside the scope of a direct replication and this investigation to try and address weaknesses or potential issues with the original research or address generalizability. These are important to address, yet we cannot expect replications – already extremely rare in the literature – to address all the challenges that we are facing in science. However, we acknowledge that there are issues with the original study that are worth documenting so that if others were to plan doing similar studies from scratch today, they could approach it differently and learn from insights we gained from the process.

In addition:

1. Direct replications address some aspects of generalizability by examining the same methods using a different larger more-diverse sample 3.5 decades after the target article's publication.
2. The impact and citation rates for the target classic article coupled with issues we document (e.g., small samples) and the lack of independent direct replications raises the

importance of revisiting and reevaluating this research. Conceptual replications are important yet make more sense once we verified that the original findings seem to hold.

3. There have been many conceptual replications in follow-up research that have demonstrated the generalizability of the paradigm of outcome bias to other contexts. What is more urgently missing is revisiting and validating these findings with independent pre-registered well-powered open materials/data/code direct replications .

Beyond this, I am positively predisposed towards the manuscript, but think it requires some work to get to the finish line. My main points of feedback, which will be further outlined below:

- **Your abstract can be much clearer**
- **Your introduction can be much shorter and much more succinct.**
- **Your pre-registration should be better organized. As an outside reader, it is unclear which document I should look at and how the three main pre-registration documents differ (note some convergence from Ropovik and myself here, see my explanation below).**
- **Ropovik is better I doing analyses than I am, so he found it easy to find the scripts and run it out of the box. For people like me, you need to give more guidance (again, see below).**
- **A major, major point: your data was not properly deidentified. Before you are going to do anything about this project and independent of whether you choose to resubmit to IRSP, you really, really need to properly deidentify your data. Your data was posted with ip address, location data, and demographics! The data are not super sensitive, but this does violate research ethics. I will remind you that IRSP is based in Europe and this would be a flagrant GDPR violation.**

Thank you, we are glad for the recognition of the value of the paper. Below, we addressed the points raised. We removed all identifiable information from the data files.

So far, I have not yet touched the discussion. You will see that I have a number of points that may change the nature of the discussion. Below, I will outline any additional points I have and I will include some points of convergence/divergence with Ropovik. I will however ask you to address all points, including Ropovik’s points that I do not mention.

We will discuss our changes to the Discussion section in the revision in subsequent points. Briefly, we added a paragraph at the beginning of the Discussion section and the Limitations section addressing issues with the original study and its generalizability.

Abstract:

- **For the abstract, I think there can be a slight language change to make it a bit clearer. At present, you write the following: "We found support for an outcome bias with stronger effects than in the original, and even for participants who stated that outcomes should not be taken into consideration when evaluating decisions. In an extension, we found differences, dependent on outcome types, in evaluations of the perceived importance of considering the outcome, the perceived responsibility of decision makers, and the perception that others would act similarly given the choice by outcome type."**
  I would make a slight change, writing something like the following: "For the replication part of the study, we found support for an outcome bias with stronger effects than in the original, and even for participants who stated that outcomes should not be taken into consideration when evaluating decisions. For the extension part of the study, we found differences, dependent on outcome types, in evaluations of the perceived importance of considering the outcome, the perceived responsibility of decision makers, and the perception that others would act similarly given the choice by outcome type."

Thank you for the suggestion. We changed to the wording in the abstract.

- **For the abstract, it was not clear to me which of the hypotheses were pre-registered and which were not.**

We marked the hypotheses that were pre-registered in Table 2 with a *. We also added clarifications in the abstract to make clearer which results were pre-registered.

- **In addition, in the abstract, you indicated to have tested "outcome bias". However, for a naïve reader, it is not clear what the actual research methods were and what you found (concretely).**

- **It is good that you mention your sample, but I would also mention the sample size of the original.**

- **I would make it clear that you switched from within- to between participants in the abstract already.**

We made changed to the abstract to address all these points.

Introduction:

- **Overall, I feel that the introduction can be much more succinct. See below for some suggestions.**
- **You deviate quite far from the original study by going into outcome bias in science. While I see its (broader) relevance, in the introduction I strongly prefer to learn more about what the underlying mechanisms are, what the original effects were, and what the shortcomings of the specific study were. The tangent about outcome bias in science more general better fits in the discussion. While I am very positively predisposed towards Registered Reports, the introduction of this manuscript is not the right place to discuss them. I think you can briefly, in one paragraph, indicate how the Baron and Kenny study influenced the rest of the literature (which you do on Page 6-7, where you can add one sentence on science). The focus, however, should be on the justification on why to replicate this work (which you do, but could be slightly more explicit).**

We moved the wider influence of the original study to the Discussion under the subheading "Broader Significance of Outcome Bias". There's quite a lot of useful context here that we feel is worth including for reader to make clear how much research has been built upon the original findings of Baron and Hershey.

- **You indicate that you want to "obtain more current effect size estimates". What does that mean? Do you expect a change over time and that because of historical changes, the effect size is smaller or larger? Or is the original sample too small to get a realistic estimate? (the general sentence: "Hence, we felt it important to revisit these findings to assess their reproducibility and replicability, and to update these findings to obtain more current effect size estimates and examine robustness to support and inspire future research." Could be split up more, unpacked better. For instance "examine robustness to support and inspire future research" What does this mean? Do you simply want to see whether the findings replicate, given that it has been such a central effect in the literature?)**

We rewrote this part in the section "Chosen Study for Replication…" in order to emphasize that the original study's sample size was fairly small and that replicating this effect with a well-powered sample would benefit subsequent research.

- On page 8: you now introduce the study (from "We….1000 times"), even though we have heard about this before already on page 5. I think you can significantly shorten your introduction by merging the information on page 8 to where it is first introduced (page 5). Similarly so, the paragraph "We found the target article…to a within subject-design" can already be integrated when you first introduce the target article. Overall, this would mean you will have one or two paragraphs on the current study and the justification for choosing this study and then its impact on the broader literature.

We now include this information in the section "Chosen Study for Replication…" and the introduction is now more concise.

- The classification from Lebel et al was not really clear to me. What are the decision criteria for classifying something as close versus distant? The classification seems a bit arbitrary to me (e.g., if one factor changes, can we easily classify as a conceptual replication?). (in general, the deviations from the earlier method can be best described in the method section)

We added a subsection entitled Replication Closeness Evaluation to explain how we arrive at this classification using the criteria from LeBel et al. (2018) and a Deviations subsection to explain differences between our replication study and the original.

- The paragraph "Recent years…in the field" can be deleted entirely. It does not contribute to the current study.

This paragraph has been deleted.

Pre-registration:

- **I arrived here, because to me in the abstract it was not clear what was, and what was not pre-registered. So I went to the pre-registration to better understand this. On your OSF page, I first go to "registrations" (https://osf.io/knjhu/registrations). I then go to the sole registration available (https://osf.io/czha8). You indicate there "Please see pre-registration folder for full pre-registration materials based on pre-registration template together with Qualtrics survey file". I then go to OSF Storage (https://osf.io/czha8/files/osfstorage), which permits me to go to "Archive of OSF Storage" and then "Pre-registration". I presume that is the correct location. There, I find five files (all uploaded the same time): A Qualtrics file (.qsf), a word export of the Qualtrics file (so far so good), but then three word files, all three with hypotheses.**

For an outside reader, it is not clear which file to rely on. I am very willing to give you the benefit of the doubt, but on your OSF page, the reader will need to have guidance what it is you actually pre-registered. It is entirely possible that the three word documents have large overlap, but I think you will understand that it is not up to the reader to organize this for you. As a reader, it would help me if you would consolidate the documents and outline the pre-registered hypotheses + power analysis.
(later note: after reading the abstract, I get to "We therefore pre-registered both together, aiming for addressing the most conservative combination of the two." Perhaps this is what you mean with the different documents on the OSF, but that should be clarified and it will help to have a consolidation on the OSF page)

Yes, these are the right files and location. Thank you, this helped us realize we could have been clearer about that aspect.

Open pre-registrations allow for a directory to be frozen to reflect the work that has been invested in constructing the pre-registration. In this case, as you've indicated, two files relate to the Qualtrics survey file, and the three files reflect work done by two different authors.

Our aim here was to crowd-source the pre-registration by having two coauthors separately analyze the target article planned analyses (similar to what we've later that year saw from the Many Analysts projects). We felt that this design increased the possibility that the combination of the two pre-registrations will yield the best overall analysis, and were aiming to address both in that we address the strictest most conservative combination of the two. When two analysts work on the same project together rather than independently they may tend to converge on something less optimal than having a free mind not being aware of the other's work. There were

some minor divergences between the pre-registrations that we noted in Table 3 in the revised manuscript (now referred to from Table 2), which also links to the specific frozen OSF files pre-registration files.

Methods

- **I don't really think that the shift from a within- to a between-participants design is a small shift. I agree with you that it is superior, but it is not a close replication. It is a considerable difference and that should at least be recognized as being a conceptual, not a close replication. I still think it is a worthwhile study, as I think improving methods should be part of replicating work.**

We understand the concern. This seems to reflect somewhat subjective taste which is worth debating, yet right now under the LeBel et al. criteria this does not seem to matter much. We added a note with a reference to a similar replication project we completed that changed from a within to between-participants design:

> We note that other replication with similar adjustments from a within-participants to a between-participants design have been classified as a "close replication" (Jamison et al., 2020).

However, to address your comment, we decided to accommodate both by summarizing this as a "close to far" replication:

> Many of the features were the same or similar, which we felt warranted the categorization of "close" and yet the evaluation criteria we used made no reference to the impact of shifting study design from a within-subject to a between-subject design, and so to accommodate for that change we summarized that as "close to far".

- **I agree with Ropovik that I was struck by how few participants were excluded and how few participants guessed the hypothesis. I went into your survey, and see you chose not to do a funneled debriefing, as is common in these kinds of studies. I would at least mention that as a shortcoming in your discussion. In addition, I think it can be useful for the reader to know how you established participant awareness. Did you have two coders code this?**

This is typical in many of the replications we conducted. The design was a between-subject design where participants are exposed to a single vignette in a very short (1-2 minutes survey) survey with online labor market participants who work under time pressure.

It is extremely difficult for participants to generally guess what the purpose was, and they seem to have no interest in trying to or reporting that they have. We note that even in with-subject

designs, it is documented that it is very difficult for participants to guess the purpose of the studies. Citation examples:

- Lambdin, C., & Shaffer, V. A. (2009). Are within-subjects designs transparent?. *Judgment and Decision Making*, 4(7), 554-566.
- Aczel, B., Szollosi, A., & Bago, B. (2018). The effect of transparency on framing effects in within-subject designs. *Journal of Behavioral Decision Making*, 31(1), 25-39.

I am reading your survey flow from the word document, but do I understand it correctly that you did not randomize order of the question within each block? (in this case, it does not seem to be a big deal, but it would be good to mention)

We made it clearer at the start of the Methods section that the order of questions was fixed for all participants.

- I personally like a power analysis before reporting participants, but you see Ropovik's and my divergence there. I am fine with either.
- Ropovik makes another important point: "Any post-treatment exclusions may easily introduce bias and are therefore a risky endeavor (see Montgomery, Nyhan, & Torres, 2018)". I agree with him. In this case, it seems to matter little, but I think it is good to make a note of this in the discussion, and I would include the participants who indicated not to have been serious in the main analyses (and make a footnote that you deviated from your preregistration, and why).

We moved the discussion of participants to before the power analysis in the Participants section. Thank you for the point on exclusions. As we preregistered the exclusion of non-serious participants, we have decided to stick with that for now. Given this is also a low number of participants, we do not see the need to deviate. We do also have analyses run with or without exclusions and have shown that there is little difference in results so this also addresses this point.

- I also agree with Ropovik that demand characteristics could have been introduced by the comprehension checks. If the comprehension checks would not have been there, the effect may have been weaker. That is not a criticism that would pevent potential publication, but I think you can make a mention of how to design a future study to investigate this.

We added a paragraph to our Limitations section to address this. Overall, the comprehension checks are probably worth including to ensure attentiveness and more research is likely needed to support the effect being a result of increased salience via comprehension checks. In similar

instances when we received peer review criticizing the use of comprehension checks in a replication as potentially resulting in the failed replication, we addressed it with a follow-up data collection randomizing the inclusion of comprehension checks between-participants, and showed that they do not seem to impact the results (e.g., Ziano et al., 2019; https://osf.io/h82s3/; Study 3b, page 24). We still prefer to include comprehension checks because including those addresses situations of failed replications in which reviewers sometimes claim that the participants did not attend to or understand the scenarios, and the comprehension checks directly address that concern.

Power Analysis and Analysis Approach

- **In Table 2, you outline 8 hypotheses, of which 5 are pre-registered (note, based on your own wording in the manuscript, because the three pre-registration documents need to be consolidated first). For the analysis section, I would therefore expect a confirmatory results section (split up between replication results and extension results) with the 5 pre-registered hypotheses and an exploratory results section with the 3 exploratory analyses. I did not see this separated.**

We now restructured the Results section into two main subsections to make clear what results are confirmatory and exploratory results.

- **Relatedly, when I go to your power analysis description in the participant section (which I think is the good section), you write the following: "We determined that at least 239 participants were needed to achieve 95% power to detect the minimum effect of dz = .21 with an alpha of .05 for the smaller effect of the two contrasts". Where is the analysis for this? What was the design for this? How can I reproduce this? Did you do a simulation in R? Was this in G*Power? Do you have the screenshot of the analyses if the latter (I imagine you ran a simulation for the mediation analyses)? In addition, as you pre-register 5 hypotheses, why did you not correct the alpha level? (notably, because you collected many more participants, you may have enough participants to test these 5 hypotheses, which you can test in a post-hoc sensitivity analysis).**

The power analysis described here is in our Supplemental Materials. We previously did not make an explicit reference to the supplementary materiasl, which we now added to this section in the Methods.

- I tried to go to your OSF page to find the data and the analysis code, to rerun the analysis. It was not clear to me which files I should take. This can be much better organized (eg one can add a readme file to guide the reader through the analyses). What I personally like is a zip file with an rproject file so that the analyses can be run out of the box (here you see a difference between Ropovik and myself again, probably driven by the fact that he is better at this, but you will have more readers like me).

A readme file has been added to the OSF 'Data and code' folder in order to explain the various files.

- I do see that you posted data with ip address (!), location data (!), demographics (eg https://osf.io/4n6hu). Data should be properly identified.

We removed these fields from the files.

Results:

- To what degree were the four items independent? Did you conduct a factor analysis? Did you look at the correlation between the items?

We added a correlation matrix to the supplementary materials, which show some weak to moderate correlations between items. This is to be expected, and this is mentioned in the manuscript.

- You did not test for any of the assumptions of the ANOVA (were the data normally distributed? Do they have a common variance? When I look at the SDs for positive versus negative outcomes, this may not be the case. If this were the case, you would have to use Welch's ANOVA instead).

The statistical functions we used in R checked for assumptions automatically and applied the appropriate test. Our t-tests, for example, were automatically applied as Welch's T-tests which explains the dfs you see (when using the t.test function in R). We made that clearer in the revision.

- "We found support for a main effect of decision maker over perceived decision quality ($F(1, 688) = 4.73$, $M_{diff} = .20$, $p = .030$" – if the alpha would have been corrected (as should have been the case), it seems this is no longer significant.

We now added Bonferroni p values for all ANOVA results after the regular p values so they can be seen before and after the correction. Given the relatively strong effects, corrections had little impact on the findings.

- **For the replication, it would help to have a table with the original results (including the effect size and its confidence interval, as well your results with the effect size and the confidence interval of the effect size). In that case, many of the results would be in the table, and in the text you verbally describe them (in the table, I would incorporate the equivalence test; in the introduction, you make no mention of this; was this pre-registered? If not, it goes into the exploratory results section).**

We added these in Table 5.

- **It's not clear which analysis you run here: "However, physicians' decisions were evaluated as lower quality than patients' decisions (t(338.62) = -2.91, Mdiff = .86, p = .004, Cohen's d = -.31, 95% CI [-.46, -.16])" As the df is a lot smaller, did you split the sample to run these analyses? Why not analyze them via contrasts (and thus rely on the variance from the entire sample)? (I see now as well that you use Welch's t-test; it would be useful to indicate this early on, as your dfs are uncommon for a regular t-test)**

We now make it clear that we used Welch's t-tests.

- **"We found that people who self-reported that they should not consider the outcome did in fact show an outcome bias (t(38.64) = 2.23, Mdiff = .75, 95% CI [.21, .1.08], p = .03, Cohen's d = .64)." again, with an alpha correction this is likely not true.**

Addressed above.

Other points:

- **I don't feel strongly about this, but it seemed that Gilad Feldman did most of the work according to the CRediT taxonomy. Why was he chosen as last author and not first author? (it can be good to briefly explain this in an author note, I imagine other readers will have the same question).**

We aimed to be very transparent about the contribution of each author and provided all details about the contribution of each author in the Authorship Declaration and CRediT table, and have worked to make things even clearer in the revision. Gilad Feldman is the coordinator of this project and served as guiding the other authors in their work, which in this and across all similar projects by this team is recognized as last position corresponding author. Gilad guided all steps in this project, yet did not do most of the work.

- **Please change language as “between-subjects” to “between-participants” (I buy into this style guide: https://owl.purdue.edu/owl/research_and_citation/apa6_style/apa_formatting_and_style_guide/apa_stylistics_basics.html - I think using “subjects” to refer to participants refer to a more passive research participant that is “subjected” to whatever the researcher would like them to do).**

This has been changed throughout the manuscript.

- **“detection of Cohen’s d = 0.25 (one-tail), considered a weak-effect in social psychology” I suspect you base yourself on Cohen’s standards, but a) I don’t think one can easily generalize effect sizes across different subdisciplines in social psychology (different methods/concepts require different thinking about effect sizes) and b) those standards are highly outdated.**

We removed the reference.

- **How did you measure “2) low English proficiency”? (I don’t think I saw it in the survey, see also Ropovik)**

- **“After excluding participants who did not complete the survey” if they missed one question, were they excluded?**

We added an explanation in the Participants subsection that we ran this study in a combination with a few other unrelated studies, presented in random order, and the question about English comprehension was shared among those studies.

Small errors:

- **“Gilad led supervised each step of the project”**

- **“We discuss future direction” should be “We discuss future directions”. However, in general, I advise against such language. Saying that you discuss future directions can better be replaced by which future directions you discuss.**

- **“main effect of outcome type.” period too many.**

These have been addressed.

Response to Reviewer #1: Dr./Prof. Ivan Ropovik

> **1. I am wondering what were the motivations of the original authors to use within-ss design, especially given the quite self-evident anchoring/carryover issue. A sentence or two on that matter may cast some light.**

We added an explanation to the introduction.

The original authors wrote the following:

> "within-subjects design makes it easier to distinguish small effects from random error but at the cost of reducing the magnitude of effects because subjects may remember responses they gave to similar cases."

> **2. "We crowdsourced pre-registrations via two co-authors working independently in tackling the analysis and reproduction of Baron and Hershey's (1988) methods and analyses.". Not entirely clear. Does that mean that your team independently prepared two pre-regs, followed by some sort of reconciliation?**

We clarified this in our reply to the editor above, and added Table 3 addressing this issue. In short, we addressed the combined strictest criteria of both.

> **3. Participants: I think starting with the description of the present sample is a more customary way of reporting. I would move the power analysis at the end of that part.**

We changed the order in Participants subsection has.

> **4. Power analysis: Power should not ideally be computed for a single arbitrary point (past result) but for the smallest effect of interest, followed by a range of hypothetical effect sizes. Ideally, the reader should be informed what power does the present design provide to detect a wider range of effects. You may even consider including (at least into SMs) the power curve. G*power that you are using has that capability. Powering to a past result (actually, to any empirical estimate) is a suboptimal practice. It is perfectly defensible to establish sample size on pragmatic grounds and descriptively inform the reader about the informativeness of the present design.**

We added a citation to Simonsohn (2015) to explain the x2.5 rule-of-thumb adjustment.

5. I see you are using one-tailed test (except for the equivalence test). It is perfectly fine in some analytic situations, especially if it was preregistered. With your N, however, using alpha of .05 with a one-tailed is too much liberal, and unnecessarily so. Also, it prevented you from meaningfully interpret a result in an opposite direction. That didn't happen but would you be fully comfortable with interpreting a strong effect in the opposite direction as consistent with H0? Yes, the original authors used a one-tailed test and that proved to be a lucky decision given that the focal effects ended up virtually at p = .05, but that is far from empirically robust. Just a thought for next research – obviously you won't change the inferential criterion ex post (and it wouldn't have any impact). But you may want to consider reporting the power curve for a two-tailed test too.

We appreciate the feedback. Yes, p-values are sensitive, which is why we supplement them with effect sizes + CIs. Given that this is a replication, and we have a clear confirmatory hypotheses in a particular direction, one-tail test seemed most relevant.

6. The exclusion criteria are not clear. What does it mean "failure to complete the survey" exactly? Low english proficiency?

We added more details on the exclusion criteria in the Participants subsection.

7. Any post-treatment exclusions may easily introduce bias and are therefore a risky endeavor (see Montgomery, Nyhan, & Torres, 2018). Conditioning on the e.g., lack of seriousness check may open a non-causal path from the experimental treatment to the outcome if there is a shared, unmeasured cause of both, participant's lack of seriousness and the outcome (i.e., carelessness). Ideally, such exclusions need to be done only as a sensitivity analysis and interpreted cautiously. In this particular case, the nature of the intervention should probably mitigate such bias but still, it is a thing to keep in mind in future research and always a lurking threat :)

Thank you. We reported findings with and without exclusions, exclusions were minor, and they had no impact on the results. We also address this in more detail in our reply to the editor.

8. Were there no missing data? If so, what feature of the design prevented missing data (and were there any consequences to the validity of measurement?). If there were missing data, please describe and tell the reader how they were handled.

We added an explanation in the Participants subsection that all questions were forced response and so there is no missing data.

> **9. I was struck with how few participants were excluded. Only 2 did not complete? So few participants correctly guessed the hypothesis of the study? I think that warrants a check. Also, please describe in more detail how the incentives were set up?**

This is fairly typical in our replications. The survey is very short (1-2 minutes), and using a between-participants design, and so the likelihood for dropping out in that short time and for participants to guess the hypothesis is very low. The numbers we included were participants who passed consent and began this specific study, included in a series of other unrelated studies in a single data collection (see reply to editor above).

> **9. Just a thought – if the decision making on probabilistic outcomes is carried out under perceived information asymmetry, outcome bias on the part of the observer may not be entirely irrational. People have the experience that medical decision-making is usually done not only based on base rates. Perceived rationality may thus overestimate the conceptual effect even if substantial proportion of participants is aware of the outcome bias issue. On the other hand, there is a counteracting demand characteristics for these people, which makes it difficult. So although the general principle that it is rational to judge decision at the time they were made, this may be a loaded example, IMO. Amateur speaking. But maybe it may be worth reviewing arguments of authors studying outcome bias in other domain for and against the specific medical example. Apart from that, the medical setting also creates the specific ethical issue you talk about in the limitations. I get that in a replication, you don't do anything about these things but a discussion of those may be interested for the reader to appraise the merits of this setting for interpreting the general principle of outcome bias.**

Thank you. We added a reference to the Bar-Hillel paper on base rate neglect at the end of the subsection on the Broader Significance of Outcome Bias. We think this makes medical context a decent one to study outcome bias in, though we admit this is only one context, with others explored in this section.

> **10. The demand characteristics effect for people aware of the outcome bias issue may have been amplified by the comprehension checks, no? That may be one of the contributing design-related reasons, you found such strong effects. Just a speculation, though.**

We added a paragraph to our limitations subsection on the potential effect of comprehension checks.

11. Typo: What percentage of people

Addressed.

12. Results: Why 90% CIs? For those Cohen's f, I would also – for the purposes of presentation – report also the Cohen's d equivalent. E.g., some readers may not easily grasp the magnitude of an effect like f = .47 – quite large.

We added a reference to Lakens (2013) to the start of the Results section for why this is the case.

13. In some instances, you interpret a non-significant result as affirming the null, which is formally not correct.

We changed the language around discussing effects to either show support or not for an effect.

14. Figures are of low quality – probably a technical glitch, but it needs to be taken care of.

We reexported the figures with a higher dpi.

15. The figures also contain BF analyses – no mention of that in the method. Probably a short paragraph describing those would be nice. And also their results – the reader would benefit if you at least briefly lay out the comparative strength of evidence in favor of a given hypothesis.

We added those to the first figure caption.

15. I get that the effect size in Baron & Hershey was d = .21. But what is the rationale behind setting the TOST lb and ub to -.21, and .21, respectively? The problem of evaluating whether effect replicated is a notorious one but why .21. Is it about crossing the 0? If so, please state that explicitly.

16. "Thus, given the power of the present study, compared to the original experiment, the outcome bias effect may be stronger than originally estimated based upon the original Baron and Hershey (1988) experiment." –> Power has little to do with the effect size under 0 publication bias – which you make the reader assume by your pre-reg. I would reformulate.

We added an explanation for why these our bounds to the subsection "Comparing replication to the original…". We think that by using the original effect sizes as our bounds, we address an interesting research question regarding whether the original effect size estimate holds.

> **17. Discussion: "We found support for outcome bias and with much larger effects (patient: original d = .21, replication d = .75," –> The numeric value here (.75) and in the results differ slightly. Please check.**

Thank you for spotting this, this was wrong in the discussion. We corrected this oversight.

> **18. Last but not least – the discussion contains several interpretations of the underlying phenomenon based purely on the very narrow operationalization of it by the original (and your) study. I would suggest a more measured interpretational stance – see the Generalizability crisis paper by Tal Yarkoni. For next replication projects, I suggest the extension may better go in the way of not having a single, fixed, very narrow stimulus, but randomizing across a universe of various design features that may be relevant for the given underlying effect.**

We now caution about this at the start of the Discussion, making reference to Yarkoni. We offered some potential rationales behind findings whilst acknowledging that these are narrow results.

> **To conclude, despite the above-mentioned, this is a very nice, well-written replication report.**

Thank you, both, for the detailed, careful thoughts on our manuscript. Your diligence and hard work is much appreciated! And we believe that the manuscript is much better as a result of your reviews.
