# Supplementary material for: Outcomes Affect Evaluations of Decision Quality: Replication and Extensions of Baron and Hershey’s (1988) Outcome Bias Experiment 1
Source: Int Rev Soc Psychol. 2023 Jul 28;36:12. doi: 10.5334/irsp.751 (PMC12372742; doi:10.5334/irsp.751)
Supplement: Review file 2. — Open Review Response for the 2nd round of review, including Response to Editor and Response to Reviewer. [file irsp-36-751-s3.pdf]

Reply to 2nd R&R decision letter review: Baron and Hershey (1988) replication and extensions

We would like to thank the editor for the useful suggestions and below we provide a detailed response as well as a tally of all the changes that were made in the manuscript. For an easier overview of all the changes made, we also provide a summary of changes.

Please note that the editor's and reviewers' comments are in **bold** with our reply underneath in normal script.

A track-changes comparison of the previous submission and the revised submission can be found on: https://draftable.com/compare/hYfqfKwyPpOb
and file "IRSP-RNR2-BH1988-rep-ext-main-manuscript-v3-G-trackchanges.docx"

Summary of changes

Below we provide a table with a summary of the main changes to the manuscript and our response to the editor and reviewers:

Section	Actions taken in the current manuscript
General	
Introduction	Minor wording changes.
Methods	Minor changes to how the study procedure/design is explained.
Results	NA
Discussion	Bulk of the changes here: section has been shortened with some references removed and extra information added on generality.
Reporting	NA
Supplementary materials	NA

Response to Editor: Prof. Hans IJzerman

> **I have now received another review from Ivan Ropovik. Before reading his review, as is my custom, I read your revised manuscript independently. Before getting to my (final) comments, let me compliment you on a nice revision. I knew it was a lot of work, and I am grateful for the work you have put in so far. Both Ropovik and I have a few remaining comments. The below seems like an extensive list, but that is because much of it is sentence-level feedback. Don't be scared off by what I provide.**
>
> **Before getting to my comments, I would also like to express that I enjoyed seeing the table and description of the two sets of pre-registrations. I hope at some stage you will work out further – in a different paper - the idea of crowd-sourced pre-registration and the way to synthesize these. You will have an interested reader in me.**
>
> **Then, the issues still to be addressed. These are comparatively minor, and I think they are all easy to fix. I went through your decision letter, but I will not address the issues that I think are solved (I am not ignoring them, but I am satisfied with how you addressed them). Beyond the issues below, please also see Ropovik's comments.**

Thank you for the positive feedback, the review obtained, and your supportive and constructive review.

> **Issues from the previous round:**
>
> 1. **I would still add in the discussion that better way to probe awareness could further improve the study (even if the funneled debriefing has its shortcomings).**

We added your suggestion on randomizing the order of comprehension checks to the "limitations and future directions" section:

> Future work could address this by checking awareness of the stimuli by other means, such as randomizing whether the comprehension checks are shown before or after participants provide their responses.

2. **“We still prefer to include comprehension checks because including those addresses situations of failed replications in which reviewers sometimes claim that the participants did not attend to or understand the scenarios, and the comprehension checks directly address that concern.”**
   à of course, an easy way to fix this is to randomize order (comprehension check first, then dv = order 1; dv first, then comprehension check = order 2). I think for future reference this is an easy fix if you want to keep comprehension checks in. No need to further address this, just a suggestion from my end for your future studies.

Thank you for the comment and suggestion. This is a valuable discussion to have, and we agree that it would be worthwhile to have an exhaustive empirical investigation on whether and to what extent comprehension checks have an impact.

As pointed out in our previous reply, we have implemented exactly that in several replication projects in which the editor and reviewers asked us to conduct another data collection either removing manipulation checks or randomizing their order, and the results indicated no difference (e.g., Ziano et al., 2019; https://osf.io/h82s3/; Study 3b, page 24). One example reason not to vary the order in every replication is because there are countless decisions that may or may not impact findings that can be randomized and the inclusion of each additional factor increases the complexity of analysis and reporting, adds limitations, and has implications for power. The reason to include comprehension checks rather than not, even if they might not impact the results (based on our experience), is because this way you rule out inattentiveness and lacking comprehension as an explanation for the possibility of a failed replication to address common criticism in advance. We acknowledge the limitation that by doing that, we are also creating a deviation that might fuel reasoning aiming to explain a failed replication, and so it comes down to addressing concerns regarding sample versus addressing the need for deviations that allow for tighter control.

3. **“Please change language as “between-subjects” to “between-participants” (I buy into this style guide: https://owl.purdue.edu/owl/research_and_citation/apa6_style/apa_formatting_and_style_guide/apa_stylistics_basics.html - I think using “subjects” to refer to participants refer to a more passive research participant that is “subjected” to whatever the researcher would like them to do).” à I think you made some corrections, but not all (my word file counts 15 more cases).**

We changed “subject(s)” to “participant(s)” throughout.

4. **I made a mistake in my original decision letter, which is now more visible after you improved your description of the original study and your own work (I should have reviewed this in the original paper, nevertheless). In my previous decision letter, I wrote:**
   "I really struggled with what decision to take. Let's face it, the original study was badly designed. In a modern study, one shouldn't accept studies that rely on single scenarios. I personally would not run a replication study like this anymore, but I would seek to improve the design. I would include the original scenario, but then write additional scenarios to increase the generalizability of the study", but, of course, that is what the original authors did. I apologize for this oversight (to you and the original authors!).

   You do write, however, that "To summarize here, our view is that replications should be focused on the task of repeating original studies and examining their reproducibility and replicability of findings, and so we see it is to be outside the scope of a direct replication and this investigation to try and address weaknesses or potential issues with the original research or address generalizability. These are important to address, yet we cannot expect replications – already extremely rare in the literature – to address all the challenges that we are facing in science. However, we acknowledge that there are issues with the original study that are worth documenting so that if others plan to do similar studies from scratch today, they could approach it differently and learn from insights we gained from the process."

   I would fundamentally disagree with this. Not all replications are worth running, particularly in case of bad measurement or manipulation that one can review as not worthwhile. Time and resources are limited, so it is reasonable to assess the value of a replication (nevertheless, the study actually did seem well-designed, as per above). As the original study was ok, the attention to this topic is less relevant.

   It is indeed the case that you chose to switch to a single scenario rather than multiple scenarios. While I agree that that limits potential noise from a within-participants design, it does also limit generalizability.

   You, therefore, write:
   "In this replication we focused on a narrow set of stimuli focused on medical decision making scenarios, and therefore we caution regarding the generalization of the target's and our replication findings to other

> **situations, domains, or the broader phenomenon of outcome bias (for a discussion of the issue, see Yarkoni, 2022). Replications are meant to reproduce and re-examine studies, and we contributed with a minor extension of the original's study design by adapting from within-participants to between-participants, yet this should only be taken as a first step for future tests of the generalizability of this paradigm. We considered it important to first revisit and re-examine the replicability of one of the most impactful classics regarding outcome bias."**
>
> **I would instead suggest incorporating the switch from the within- to between-participants design:**
> **"In this replication, we focused on a single scenario focused on medical decision-making with various outcomes, limiting the generalizability further from the original, 15-scenario design (for a discussion of the issue, see Yarkoni, 2022). The switch to this single scenario could have contributed to the larger effect size. Replications are meant to reproduce and re-examine studies. We contributed with a minor extension of the original's study design by adapting from within-participants to between-participants, yet this should only be taken as a first step for future tests of the generalizability of this paradigm. We considered it important first to revisit and re-examine the replicability of one of the most impactful classics regarding outcome bias."**

We would be happy to have that discussion with you and the community. It is a discussion worth having. We are currently engaged in several projects aimed at these very topics.

For this specific project, we agree that the best way would be to be clear, accurate, and humble about what we did and what we found.

Thank you for the suggested wording, we replaced our text with your suggested text in the discussion section. We appreciate that there are limitations with the original study that we inherited but can be addressed with our particular design.

Discussion and balance of topics:

5. In the previous decision letter, I indicated that I did not touch on the discussion yet, which I will do now. I think the balance in the discussion is a bit off. Of course, you have quite a few extensions in your work, which makes it difficult to summarize this in a brief section. I would nevertheless like to encourage you to do so.

I think the sections "perceived responsibility" to the end of "perceived norms" can be reduced to one page, maximum (for instance, I could see you remove the parts about regret, about the different stages of Heider's model, et cetera; there is quite a lot of new information introduced here. Please try to be strict with yourself when rewriting and see what you need for the main message of your work).
I think the main focus of your article is replication, with the extensions as a nice side benefit. That balance should be reflected in the discussion.

We removed some of the references that interpreted the extension results in the "perceived responsibility" section, such as the ones mentioned here on regret and Heider's model. That subsection now fits into a single page.

6. Similarly, the "broader importance of outcome bias" can be cut in about half. Again, I think one should be quite modest about broader implications for a single-scenario study.

Instead, I think it is worthwhile to add a "Constraints on Generality" section (see Simons et al. 2017), which limits your inferences to the scenario you used and the population you sampled (or you make your predictions explicit). This Constraints on Generality can probably be integrated with the "Broader importance of outcome bias" in some way.

Thank you.

That section was meant to be about outcome bias more broadly, to highlight some of what has been done on the topic, rather than to generalize from our own investigation and replication. We therefore made that clearer with

> "If outcome bias is a broad generalizable phenomenon that impacts decision-making and evaluations, then it may have broad implications for many domains. […]"

and

> "[…] Future replications may aim to revisit these findings to further demonstrate the generalizability and importance of outcome bias with potential interventions aimed to mitigate it."

We aimed to keep "broader importance of outcome bias" subsection with a few relevant citations to give more credit to some of the work done in this domain. We cut out some of the references, such as those to do with ethics and game theory.

We added the suggested "Constraints on Generality" subsection right after "Broader importance of outcome bias".

> **Medium-size issues:**
>
> **7. It is good to be even more precise about whether you replicated. I suggest the following phrasing:**
> **'For the replication (preregistered) part of the study, we found support for an outcome bias with stronger effects than in the original (original *d* = .21 - .53; replication *d* = .77 [.62, .93] to *d* = 1.1 [.94, 1.26])"**
> **I would write**
> **"For the replication (preregistered) part of the study, we successfully replicate significance value and direction of the outcome bias, but not effect size: we found stronger effects than in the original (original *d* = .21 - .53; replication *d* = .77 [.62, .93] to *d* = 1.1 [.94, 1.26])".**
> **I realize I suggested slightly different phrasing before, but this can clarify your case (and I think this new phrasing does justice to your equivalence test).**

Following your comment on the confidence intervals we had in the summary table we consulted with the community, and have come to realize oversights in our original submission. The general advice we receive is to not compare (or not to draw conclusions from comparisons) of effect sizes from different designs. We will explain.

First, we realized that there are many ways to calculated paired-samples standardized effect sizes, often used interchangeably under the "Cohen's d" umbrella term, yet diverge greatly in their range (example: Five different "Cohen's d" statistics for within-subject designs). Second, following that, comparisons between paired samples effect sizes calculated differently should be done cautiously. Third, following the first two notes, that comparisons between effect sizes of paired samples and independent samples are best avoided.

Therefore, we decided to make the following adjustments in our revision:

1. We decided to amend our use of the LeBel et al. (2019) paradigm to rely on signal (which you refer to as "significance value") and directionality, without the comparison of effects.

We added an explanation about that decision. In the comparison table we added the following note:

a. "We used the LeBel et al. (2019) paradigm for comparison of original and replication only with reference to signal and direction, yet with no reference to confidence interval overlap. This is because we switched the design from within to between participant, which makes such comparisons problematic."

2. When we report effect sizes and confidence intervals, we now indicate the type of effect, and add a note of how those were computed and suggest caution in comparing between effects. The table now includes references to d_{paired} and $d_{indpeendent}$. We added the following note:

   a. "In addition, the effect and CIs for the aggregate were computed using the t-values provided in the target article (given that no means and standard deviations were provided for the aggregate), whereas the effects for patient and physician were calculated from means and standard deviations (given that no t-values were provided). We note caution in comparing the two, given the many methods to calculate effects for paired-samples."

3. We amended the Abstract to remove the reference to effects and confidence intervals comparisons. We changed our use of the wording of "significance value" to "signal", the same term used by LeBel et al. (2019).

8. I think it is vital to know which other studies were done with this study. After the Many Labs studies and in the absence of further evidence, I am not of the camp that I think there are significant carry-over effects, but it is important to know which studies these are for a reader to evaluate. Can you post a link to them? (ideally, the analysis is also done with a variable "order" to see if order moderates the effects. This analysis can be in a footnote; I don't expect it to make any difference).

The data collection was completed with the following project, replications of two studies on the "Effort Heuristic" from Kruger et al. (2004), one mostly successful, one mixed:

Ziano, I., Yeung, S. K., Lee, C. S., Shi, J., & Feldman, G. (2023). "The Effort Heuristic" revisited: Mixed results for replications of Kruger et al. (2004)'s Experiments 1 and 2. https://doi.org/10.17605/OSF.IO/QXF5C

We added this reference and the following mention in the methods section mentioning the combined run (in underline):

We ran this study alongside a few other unrelated studies within the same Qualtrics survey (with the studies presented in a random order), and hence the question on English proficiency was shared among these studies (<u>specifically, with Ziano et al., 2023</u>).

Given that the effects in the current replication are inline with the original's, we do not see an obvious way of how this pattern would be explained by the inclusion of this study in the same data collection with another study. The combined replication studies data collection with randomized order helps address any concerns of sample quality or inattentiveness in case one is a successful replication and the other mixed or failed.

9. P. 12: Can you use meaningful names instead of "conditions 1 and 2"?

Thank you, good point. We changed to the following:

> We initially conducted a power analysis of the effects for the differences between conditions 1 (Physician Success) and 2 (Physician Failure) and between conditions 3 (Patient Success) and 4 (Patient Failure) of Experiment 1 in Baron and Hershey (1988).

10. Table 5 implies that the confidence interval of the effect size includes 0. Were these original results non-significant?

Thank you for catching that! We are very grateful.

It is a bit tricky to calculate effects and confidence intervals for paired-samples effects when raw data and information is missing, and there are several ways to calculate effects for paired-samples (e.g., Five different "Cohen's d" statistics for within-subject designs). We seem to have calculated the confidence intervals code using a package that employed a between-participant calculation instead of a within participant paired samples calculation.

We replaced the effect size calculations previously provided in the supplementary with Rmarkdown code and outputs using the MOTE r package.

We integrated the previous Table 5 with the table comparing the findings in the target and in our replication, given that they convey the same information, now in Table 7:

Table 7

Comparison of effects between the target article and our replication

Decision-makers	Original Effect Size Estimate (d_{paired}) and 95% confidence intervals	Replication Effect Size ($d_{indpeendent}$) and 95% confidence intervals	Replication Interpretation (LeBel et al., 2019)
Patient	0.21<br>[-0.23, 0.66]	0.77<br>[0.62, 0.93]	Signal and same direction.
Physician	0.53<br>[0.06, 0.99]	1.10<br>[0.94, 1.26]	Signal and same direction.
Aggregate of all scenarios	0.90<br>[0.37, 1.42]		

Note. The effect for the original is for paired-samples whereas our replication is for an independent samples and should therefore be interpreted with caution.
We used the LeBel et al. (2019) paradigm for comparison of original and replication only with reference to signal and direction, yet with no reference to confidence interval overlap. This is because we switched the design from within to between participant, which makes such comparisons problematic.
In addition, the effect and CIs for the aggregate were computed using the t-values provided in the target article (given that no means and standard deviations were provided for the aggregate), whereas the effects for patient and physician were calculated from means and standard deviations (given that no t-values were provided). We note caution in comparing the two, given the many methods to calculate effects for paired-samples.

We note that the t-test analyses reported in the original paper pooled multiple 'success' and 'failure' scenarios/cases together and then compared as aggregated. For example, when looking at the patient decision maker cases, they combine both the heart surgery and liver surgery cases together when conducting a t-test. They wrote the following:

> "the outcome bias was also found for just those cases (Cases 3 and 7 vs. 4 and 8) in which the patient made the decision rather than the physician: M = 0.48, $t(19) = 2.59$, $p <$ .01."

In this specific case, we compared conditions 1 and 2 and cases 3 and 4 which not analyzed separately in the original paper. It is indeed the case that was a signal ($p < .05$) only for the Physician but not the Patient.

Also, we added a row indicating our calculation of the effect size of the aggregate reported for all the effects combined. To compute that effect size, we had to rely on the t-value, which is tricky

to compare to both the single within effects Cohen’s d and the independent-samples d. Though for those specific scenarios our effects are stronger, our effects’ confidence intervals overlap with that aggregate effect size. As you can see in the table above, we added that as a note in the table.

11. P. 16: “The analysis script and data files can be found on the OSF page for this study.” - Can you add the direct link here?

We added the link to the OSF to the results section first paragraph:

> We provided the analysis scripts and data files on the OSF folder (https://osf.io/knjhu/).

12. P. 17: “We concluded that these findings may indicate a successful replication of the original experiment.”
I think one can reasonably disagree about this statement. I think you can explicate this in the discussion, that you replicated in terms of significance value and direction of the effect, but not in terms of effect size. I think the way you discuss this on page 20 and Table 8 on page 21 you set this up quite nicely, so if you return to this in the discussion, you have a nice round argument of what it means to replicate (again, if you save space on the extensions of the study, you have a bit more room to discuss something like this).

We noted some issues we realized regarding comparison of effects from different designs. The common criteria for evaluations of replications seems to be signal and direction, and to conclude anything but a successful replication because we detected larger effects would seem strange. This is especially so given that we focused on a subset of the target’s items, and that our confidence intervals overlap with the aggregate’s effect (now added to the table; though we again note the need for caution regarding any comparisons for effects from different designs/calculations).

We changed the wording to the following to try and make that more accurate:

> We concluded that these findings indicate a successful replication of the phenomenon in terms of direction and signal that supports the predictions and the aggregate findings of the original’s experiment. We observed larger effect size for the specific scenarios our replication was focused on, yet we note the need for caution in comparing effects from different study designs.

13. "It is possible that in a within-participants design, participants anchor their evaluation for one outcome type when providing a subsequent decision evaluation." à
"It is possible that in a within-participants design, participants anchor their evaluation for one outcome type when providing a subsequent decision evaluation or that the scenario we chose was simply the most impactful".
I would add here the possibility that the scenario you picked could have been different in terms of impact from the others.

This does not seem to be the case, given the findings reported in the target, yet we are open to that possibility. We changed to the following:

> It is possible that in a within-participants design, participants anchor their evaluation for one outcome type when providing a subsequent decision evaluation or that the scenarios we chose were the most impactful.

Small issues (but would still like to see corrected):

Thank you for catching all those, much appreciated!

14. I would mention the CI of the effect size here as well:
"and even for participants who stated that outcomes should not be taken into consideration when evaluating decisions"

We added effect size and CI to the Abstract:

> "and even for participants who stated that outcomes should not be taken into consideration when evaluating decisions (d = 0.64 [0.21, 1.08])."

15. "posthoc" --> "post hoc"

Changed accordingly.

16. In the abstract, you write "preregistered"; in the second paragraph, you write "pre-registered". Either is fine with me, but I would request you be consistent.

We changed the instance of 'preregistered' to 'pre-registered' throughout.

17. You can remove this sentence: "We begin by introducing the literature on outcome bias and the chosen article for replication - Baron and Hershey (1988), and then introduce our extensions and the research design."

Removed.

18. P. 5: “outcomes bias” --> "outcome bias”

Done.

19. “1000” à “1,000”

Done

20. “decision making” --> “decision-making” (please check throughout)

Done for all occurrences.

21. For this sentence: “We used a different physical setting, as the original study was conducted in-person whereas we conducted the study online with participants recruited from labor markets, and so our study population was different to that of the original study, which recruited only undergraduate students from the University of Pennsylvania” – I would recommend adding in a brief footnote, that a) the authors originally generalized to all humans, not just students from UPenn, and that b) a priori and without any theoretical predictions, it is unreasonable to assume non-replication by simply switching to a different population (you could cite ManyLabs2 for this).

We added the following:

> In the target article, the claims made were not about a specific population (UPenn students), and therefore we assumed broader generalizability to other populations.

22. “It should be noted that this study was run alongside a few other unrelated studies within the same Qualtrics survey (with the studies presented in a random order) and hence the question on English proficiency was shared among these studies.”
This sentence can become
“We ran this study alongside a few other unrelated studies within the same Qualtrics survey (with the studies presented in a random order), and hence the question on English proficiency was shared among these studies.”

We amended accordingly in the methods section.

23. This can be further clarified: "low English proficiency (rating less than 5 on a 1-7 scale)" (I presume this was self-declared proficiency?).

We added a clarification on the scale:

> 2) low English proficiency (rating of less than 5 on a self-rated 1-7 scale for the question "How would you generally rate your understanding of the English used in this study?");

24. P. 12: "multiple" --> "multiply"

Changed accordingly.

25. P. 20: "bias. As" --> "bias. As"

We are not sure we understand this feedback. The correction you suggested seems to be the same. This seems to be on page 33, but it does not seem to be a typo?

26. P. 20 "decision," -->"decision."

Fixed. This is on page 21.

27. P. 21 "vs" --> "vs." (see elsewhere as well)

We replaced all occurrences of this.

28. P. 24 "decision maker" --> "decision-maker"

Changed for all occurrences.

29. P. 26 "[-.09, .21]). Decisions" --> "[-.09, .21]). Decisions"

We are not sure we understand this feedback. The correction you suggested seems to be the same. This seems to be on page 27, but it does not seem to be a typo?

30. P. 26: "In the supplementary materials (from page 9)," à it is not clear what "from page 9" means here. Do you have a direct link to your OSF page where you post these supplementary analyses?

We now refer to the section name in the supplementary instead of page number (which tend to shift when combined with other documents and converted to a PDF):

> "In section "Mediation Analyses" of the supplementary materials, […]"

31. P. 26 (and possibly elsewhere) "i.e." à Latin abbreviations are according to APA style, followed by a comma "i.e.,"

We changed all occurrences.

32. P. 28 "decision making" --> "decision-making" (please check throughout; I stopped looking after this one).

Changed throughout.

33. "Kahneman & Tversky, 1982, Feldman & Albarracín, 2016" --> references should be in alphabetical order (please check throughout)

Corrected. We also checked other references.

Response to Reviewer #1: Dr./Prof. Ivan Ropovik

> **I think the authors did a good job in revising the manuscript based on my suggestions. What I appreciate is how the revision was nicely documented and made clear by using the draftable platform. I went through the revisions and rebuttals and I largely have two minor suggestions for the authors to consider.**

Thank you for your kind words and for your work in helping to improve the manuscript.

> **1. Using the 2.5x rule proposed by Simonsohn is an okay heuristic to provide the replication study with a rather decent chance to confront the original result. However, I was asking for something different. I, as a reader of your paper, would like to see what power does your present design (the given test of the effect on the given sample) has for a range of hypothetical effect sizes. A power curve generated from G*power will do. That will give a nice, comprehensive picture about the informativeness of your present design.**

We conducted sensitivity analyses, and provided those in the supplementary materials. We include a summary of that analysis and a reference to the supplementary:

> Our analysis post exclusion below resulted in 692 participants, and our sensitivity analysis indicated that for a between-participant design allows the detection of Cohen's *d* = 0.25 (one-tail). The sensitivity analysis and power curve are available in the Supplemental Materials.

> **2. I get your reasoning re my original point #5 regarding one-tailed test. I understand that the decision was primarily driven by the fact that you want to test the replicability of the original effect, I just thought that a 2-tailed test would do the same, but would also allow you to interpret an effect if it ended in the opposite direction (which should always be a consideration). Of course you will not change that ex post. That said, this is a rather crucial inferential decision so at least, I would expect a word or two on the choice of a one-tailed test and maybe (not sure about that myself) a reflection on the original one-tailed .05 result.**

Thank you.

We added the following to the beginning of the results section:

> In addition, Welch’s independent-samples one-tailed t-tests were used to test specific hypotheses by outcome type. We use one-tailed tests given that we had clear hypotheses and aimed to replicate and confirm clear predictions reported in the target article, though we note that the relatively strong effects in support of outcome bias hold for two-tailed tests.
